# Sensory noise predicts divisive reshaping of receptive fields

**DOI:** 10.1371/journal.pcbi.1005582

**Published:** 2017-06-16

**Authors:** Matthew Chalk, Paul Masset, Sophie Deneve, Boris Gutkin

**Affiliations:** 1 Institute of Science and Technology Austria, Klosterneuburg, Austria; 2 Department of Neuroscience, Cold Spring Harbor Laboratory, Cold Spring Harbor, New York, United States of America; 3 Watson School of Biological Sciences, Cold Spring Harbor, New York, United States of America; 4 National Research University Higher School of Economics, Center for Cognition and Decision Making, Moscow, Russia; 5 Group for Neural Theory, LNC INSERM U960, Departement d’Etudes Cognitive, Ecole Normale Superieure PSL* University, Paris, France; Technische Universitat Chemnitz, GERMANY

## Abstract

In order to respond reliably to specific features of their environment, sensory neurons need to integrate multiple incoming noisy signals. Crucially, they also need to compete for the interpretation of those signals with other neurons representing similar features. The form that this competition should take depends critically on the noise corrupting these signals. In this study we show that for the type of noise commonly observed in sensory systems, whose variance scales with the mean signal, sensory neurons should selectively divide their input signals by their predictions, suppressing ambiguous cues while amplifying others. Any change in the stimulus context alters which inputs are suppressed, leading to a deep dynamic reshaping of neural receptive fields going far beyond simple surround suppression. Paradoxically, these highly variable receptive fields go alongside and are in fact required for an invariant representation of external sensory features. In addition to offering a normative account of context-dependent changes in sensory responses, perceptual inference in the presence of signal-dependent noise accounts for ubiquitous features of sensory neurons such as divisive normalization, gain control and contrast dependent temporal dynamics.

## Author summary

Perception involves using incoming sensory signals to infer which objects or features are present in the surroundings. To do this, sensory systems must perform two basic operations: (i) combination of noisy sensory cues, and (ii) competition between different percepts. Here we show that the optimal form of competition depends on how sensory signals are corrupted by noise. Moreover, for the type of noise commonly observed in sensory systems, whose variance scales with the signal amplitude, competition should occur between different sensory cues *before* they are combined. Implemented neurally, this results in a highly flexible representation, in which neural receptive fields change dynamically depending on the stimulus context. Further we show that competition should take the form of divisive inhibition from the surround, accounting for why divisive normalisation, gain control and contrast dependent temporal dynamics appear so ubiquitous in sensory areas.

## Introduction

**Fig 1:**
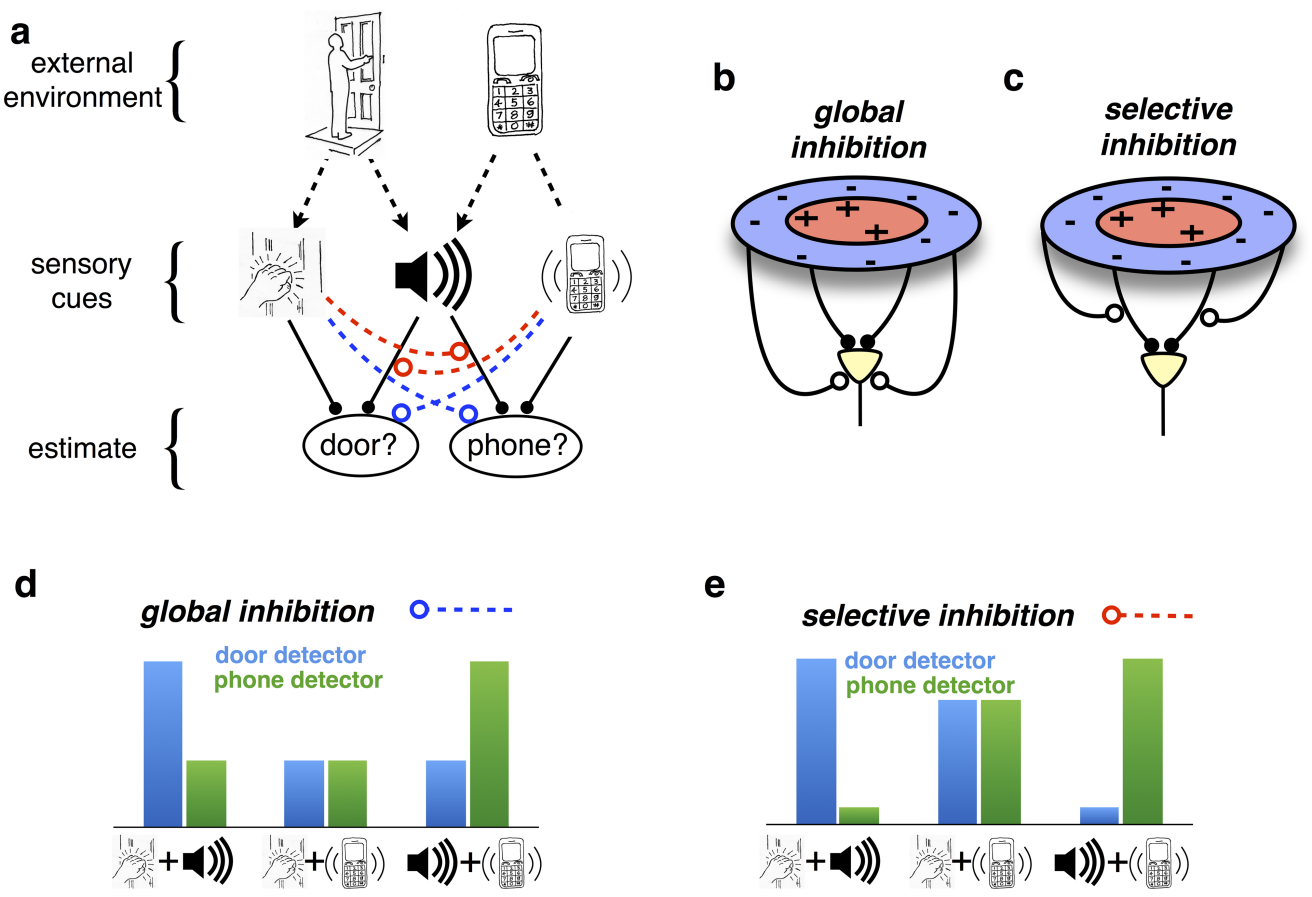
‘Explaining away’ in sensory perception. (a) The presumed goal of perception is to infer the state of the external world from received sensory cues. Here, two possible events (someone arriving at the door, and a telephone call) can give rise to three sensory cues (a knocking sound, ringing sound, or vibration). The ringing sound is ambiguous: it can come from either the door bell or the phone. Cues, such as a vibrating telephone, can resolve this ambiguity: here, increasing the chances that the phone is ringing, while decreasing the chances that there is someone at the door. Such competition between different explanations for received sensory cues is called ‘explaining away’. (b-c) In sensory neural circuits, explaining away results in suppression from non-preferred stimuli in the surround. Its effects vary dramatically, depending on whether inhibition acts (b) globally on the neural responses or (c) selectively, on certain neural inputs. (d-e) Hypothetical response of ‘door’ and ‘phone’ selective neurons, in response to different combinations of sensory cues. The qualitative effects of explaining away depend on whether it (d) globally suppresses the response of one or other detector, or (e) selectively suppresses the influence of certain cues.

A fundamental goal of any perceptual system is to infer the state of the environment from received sensory signals. These signals are generally noisy and unreliable, so that the same signal can correspond to many different states of the world. For example, the sound of a bell may mean my mobile phone is ringing or there is someone at the door. Contextual cues, such as a vibration in my pocket, can resolve such ambiguities, in this case suggesting that my phone is ringing, and not the doorbell (Fig 1a). Such competition between different explanations of sensory signals is called ‘explaining away’ and is a basic requirement for a perceptual system to discriminate between similar features. Neurally, it implies that groups of neurons which encode different (but overlapping) stimuli (such as the ‘telephone’ and ‘door’) should actively compete, via recurrent suppression [1].

The way this competition is implemented has a crucial impact on how neural responses are modulated by stimulus context.

In many ‘classical’ models of early visual processing, visual neurons are assumed to integrate inputs from within their receptive field, before undergoing divisive or subtractive inhibition from the surround (Fig 1b) [2]. In this case, non-preferred stimuli produce a general suppression of neural responses, but no changes to neural RF and/or tuning curve shapes (only a general suppression). Returning to our previous example, this would predict a general suppression of ‘door selective’ neurons when the phone was vibrating. In other words, the phone vibration would equally suppress the response of these neurons to ringing *and* knocking sounds (Fig 1d).

However, explaining away as described above requires a markedly different form of competition, with inhibition from non-preferred stimuli targeting specific neural inputs, before they are combined (Fig 1c). In this case, suppression would cause neurons to become unresponsive to certain inputs, but not others, resulting in a qualitative modulation of their receptive field (RF) shapes and/or tuning curves. For example, if the phone is vibrating, suggesting someone is calling, then the ringing sound (now explained by another cause) should not activate the door selective neurons. However, this should not affect how these neurons respond to other cues, such as a knocking sound (since the phone might be ringing whilst someone is also knocking on the door; Fig 1e).

Here, we show that the specific form that this input-specific suppression should take depends on how incoming signals are corrupted by noise. In turn, this will deeply affect the predicted dynamics and integrative properties of sensory neurons. For example, if the noise was Gaussian with a fixed variance independent of the signal strength, a sensory neuron should subtract from the other neuron’s inputs its prediction of these inputs. Because this operation is linear, the overall effect is equivalent to a global subtractive suppression by the surround (i.e. the sum of all subtractive inhibitions from other neurons), bringing us back to ‘classical’ models of sensory processing (Fig 1b).

However, sensory receptor responses and neural firing rates generally exhibit *signal-dependent noise*, whose variance scales proportionally with their amplitude [3–5]. We show that in this case, competition should take the specific form of divisive suppression, where *each individual neural input* is divided by its prediction from other neurons. Since this occurs *before* these inputs are combined (Fig 1c), it is in no way equivalent to a global surround suppression (either divisive or subtractive). Instead, the receptive fields of individual neurons are dynamically and selectively reshaped by the surround.

Divisive inhibition of each input by the surround accounts for experimental evidence showing that neural receptive fields are constantly reshaped by the spatiotemporal context of presented stimuli [6–9]. Importantly, these contextual changes in neural RFs and tuning curves do not imply variations in the stimulus features encoded by each neuron. Rather, variable receptive fields are *required* in order to maintain an invariant neural code that can be read-out consistently by downstream neurons.

Note that this framework is normative and does not depend on how it is implemented at the neuronal level. However, in order to provide more specific predictions, we show that optimal estimation can be performed within a plausible neural circuit in which excitatory neurons undergo divisive inhibition from local interneurons. Neurons in that circuit exhibit general properties of sensory neural responses including response saturation, gain modulation by background stimuli and contrast-dependent temporal dynamics. For a subclass of ‘simple’ stimuli, the responses of excitatory neurons in this network can be phenomenologically described using the canonical divisive normalization model of Heeger et al. [2, 10–14]. This accounts for why divisive normalization appears so ubiquitously across different sensory areas and organisms. It further suggests avenues for how this canonical model may need to be extended to account for the richness and selectivity of surround suppression and contextual modulation in general.

## Results

### Competition & integration in perceptual inference

To interact effectively with our environment, we need to know ‘what’s there’. Thus, perception can be viewed as an inference problem, in which sensory systems infer which combination of stimuli is the most likely, given the noisy signals they receive. Perceptual inference requires basic assumptions about how sensory signals are generated by external stimuli, which can be expressed mathematically using a ‘generative model’. Here, we consider a simple generative model, in which multiple positive stimulus features, ***x*** = (*x*_1_, *x*_2_, …, *x*_*n*_), combine linearly to activate a population of neural inputs, ***s*** = (*s*_1_, *s*_2_, …, *s*_*n*_). The mean expected response of the *j*^*th*^ input to a stimulus, ***x***, is:

$$\langle s_{j}\;\left(x\right)\rangle =\sum_{k}w_{jk}x_{k}+w_{0},$$ (1)

where *w*_*jk*_ describes how strongly the *j*^*th*^ input is activated by the *k*^*th*^ stimulus feature and *w*_0_ describes its mean activity when all stimulus features are zero. The presumed goal of sensory processing is to estimate stimulus features, $\hat{x}$, from the received input, ***s***.

Consider a population of neurons that encodes stimulus features, $\hat{x}=({\hat{x}}_{1},{\hat{x}}_{2},\ldots ,{\hat{x}}_{n})$, via their firing rates, $r\;(\hat{x})$. While the stimulus features cannot usually be estimated directly by pooling the neural inputs, we can set up dynamics of the network so that the encoded features, $\hat{x}$, converge to the most likely solution. This will be satisfied if the encoded stimulus features vary in time according to,

$$\frac{\partial {\hat{x}}_{i}}{\partial t}=\eta \frac{\partial \log p\;\left(s|\hat{x}\right)}{\partial {\hat{x}}_{i}},$$ (2)

where $p\;(s|\hat{x})$ describes the probability that a stimulus, $\hat{x}$, would give rise to neural inputs ***s***, and *η* is a free parameter determining how quickly the estimates vary in time. These dynamics ensure that the encoded stimulus features, $\hat{x}$, converge on a local maximum of $p\;(s|\hat{x})$ [15, 16].

The neural dynamics required to implement the Eq 2 will depend critically on the input statistics, described by $p\;(s|\hat{x})$. In particular, different assumptions about the *reliability* of the neural inputs will lead to qualitatively very different predictions.

A common experimental observation is that sensory neurons exhibit *signal-dependent noise*, in which the trial-by-trial variance in single neuron firing rates scales proportionally with their mean firing rate [4, 5]. When neural inputs are corrupted by independent *Poisson* noise (a paradigmatic signal-dependent distribution), Eq 2 becomes:

$$\frac{\partial {\hat{x}}_{i}}{\partial t}=\eta \sum_{j}w_{ji}\left(\frac{s_{j}}{\sum_{k}\;w_{jk}{\hat{x}}_{k}+w_{0}}-1\right).$$ (3)

Thus, the estimate of each stimulus feature varies in time according to a linear sum of ‘fractional prediction errors’, $\frac{s_{j}}{\langle s_{j}\;(\hat{x})\rangle }-1$, equal to the ratio between the received input and the mean predicted input (given the current estimate), minus one (see section 1 in S1 Text for derivation). If the received input is equal to the predicted input, then the fractional prediction error is zero, and the estimate does not change. However, if the received input is larger or smaller than the predicted input, then the estimate is updated to reduce the error.

Importantly, dividing the received input by the predicted input is necessary to perform optimal estimation given many different types of signal-dependent noise—as long as the variance in each input is proportional to its mean (section 1 in S1 Text). Poisson input is but one example of such signal-dependent noise statistics. Furthermore, while noise correlations will introduce further terms to Eq 3, these additional terms also require dividing the received input by the predicted input (section 2 in S1 Text).

We note that ‘noise’ in our model refers to trial-by-trial variability of neural inputs, *s*, given fixed external stimulus features, *x*. In contrast, the dynamics of the model network, described by Eq 3, are deterministic (see Discussion).

In Eq 3, each input (*s*_*j*_) is divided by a different factor $\left(\langle s_{j}\;(\hat{x})\rangle \right)$, before being combined with other inputs. Thus, any neural network implementation of Eq 3 will need to normalize different inputs separately, *before* they are combined (Fig 1c).

For comparison, let us consider an artificial example with the input signal corrupted by constant Gaussian noise, whose magnitude is independent of the signal strength. In such a scenario, the estimate of each feature would evolve as a function of the *absolute* (rather than the ‘fractional’) prediction errors, $s_{j}-{\hat{s}}_{j}$. Eq 3 could then be separated into two linear terms: a feedforward input and a subtractive lateral inhibition term (see Methods). Moreover, steady neural responses could be described as applying a ‘center-surround’ feedforward receptive field to the stimulus. Thus, if sensory noise was constant Gaussian and not signal dependent, competition between encoded features would result in a global ‘inhibitory surround’, separable from a static feed-forward ‘center’ (Fig 1b).

In the rest of the paper we refer to the network assuming constant Gaussian noise as the ‘subtractive model’, as opposed to the model assuming signal-dependent noise, which we call the ‘divisive’ model.

### Reshaping of sensory receptive fields and tuning curves by the context

To relate the estimation algorithm described in the previous section to neural data, we make the basic assumption that each neuron encodes a single stimulus feature, with firing rate proportional to the estimated feature ($r_{i}\propto {\hat{x}}_{i}$; see later for neural implementation).

The divisive model described by Eq 3 requires selective inhibition of specific neural inputs, before they are combined. Thus, if certain inputs are predicted by the stimulus context, they will be inhibited, and the neuron will become differentially less responsive to them. As a result, a neuron’s stimulus selectivity will be reshaped by the context. In contrast, in the subtractive model (see Methods), inhibition acts globally to alter the magnitude of neural responses, but not their stimulus selectivity.

**Fig 2:**
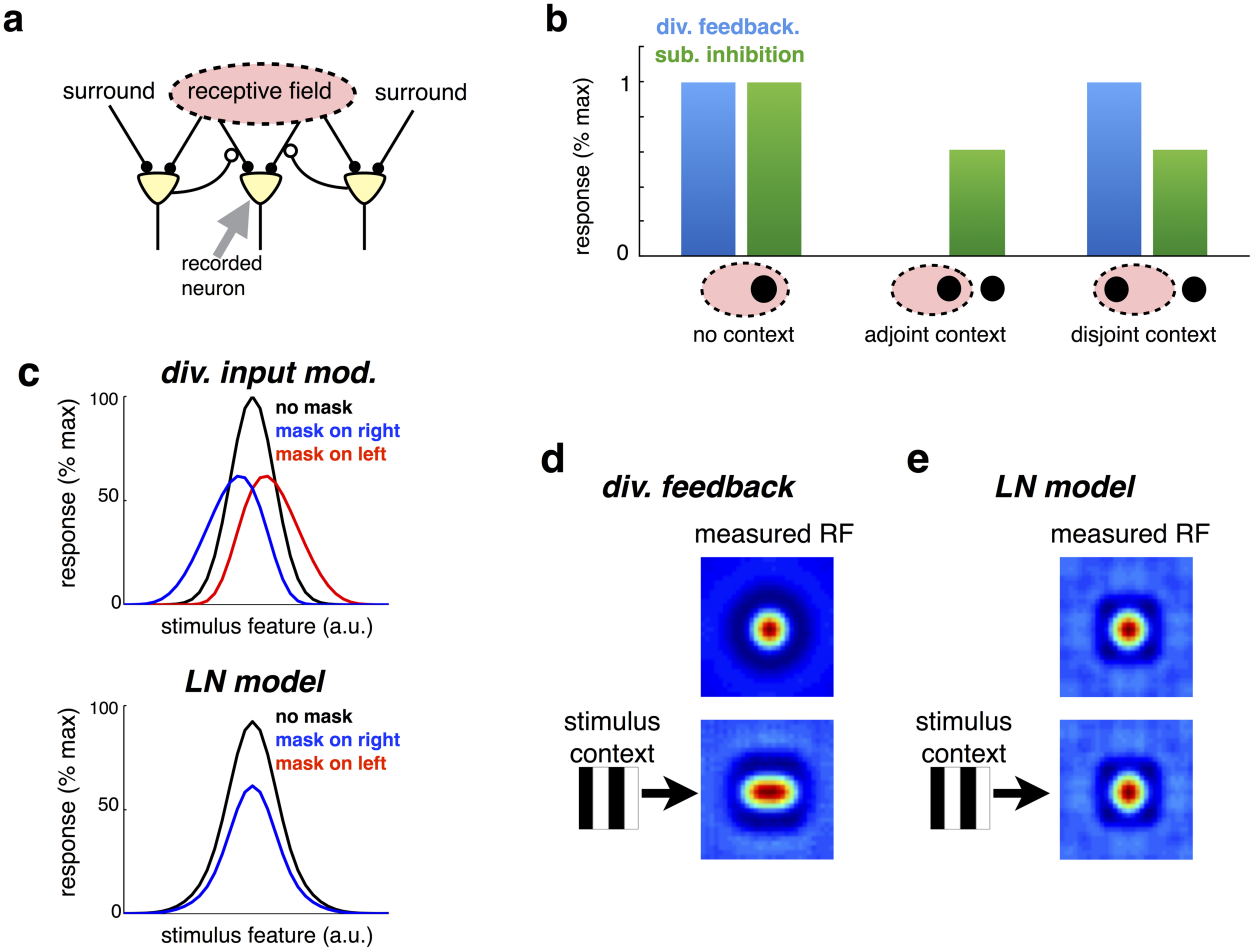
Input-targeted inhibition alters neural selectivity. (a) Schematic of neural network, with input-targeted feedback. (b) Steady-state response responses of recorded neuron, predicted by model with input-targeted divisive feedback, or subtractive inhibition. There are three stimulus conditions: (i) ‘no context’ condition, with a single stimulus within the cell’s RF; (ii) ‘adjoint context’ condition, with a second stimulus in the surround, near to the stimulus within the RF and (iii) ‘disjoint context’ condition, with a second stimulus in the surround, far from the stimulus within the RF. (c) Contextual shifts in neural tuning curves. Each neuron encodes a stimulus features (e.g. orientation, or motion direction) with a given preferred value. The mean response of a single neuron is plotted against the presented stimulus value, in the absence (black) or presence of an overlapping mask, to the right or left of the neuron’s preferred stimulus (blue and red). (c, lower panel) As above, but for an LN model. (d) Simulation of network in which cells encode stimuli in a circular region of space. (top panel) Estimated RF, with random sparse stimulus. (lower panel) Estimated RF in presence of vertical mask. The measured RF is elongated in the horizontal direction. (e) As for panel d, but for an LN model.

To illustrate this, we first consider a simple generative model, where each stimulus feature is assumed to activate two neighbouring sensory inputs. This results in the network shown in Fig 2a, where each neuron receives two equal strength inputs from neighbouring locations in the previous layer. With both subtractive and divisive models, each neuron responds equally strongly to both its inputs (‘no context’ condition; Fig 2b), while being suppressed by contextual ‘surround’ stimuli, that do not elicit a response when presented alone. However, in the divisive model inhibition selectively targets certain inputs, so that a surround stimulus only suppresses a neuron’s response to *nearby* inputs (that are ‘predicted’ by the surround). As a result, neurons respond less strongly to stimuli presented in parts of their receptive field that are near the surround (‘adjoint context’; Fig 2b), than to stimuli presented far from the surround (‘disjoint context’). In contrast, the subtractive model predicts the same degree of surround suppression, regardless of the location of stimuli within the cell’s receptive field.

A further and related consequence of input-targeted inhibition, is that neural tuning curves are reshaped by contextual stimulation. To illustrate this effect, we considered a generative model in which stimulus features activate nearby sensory inputs, arranged along a single dimension (e.g. representing the orientation of a presented visual stimulus). In the resulting network, neurons responded with bell shaped tuning curves to presented stimuli (Fig 2c, top left panel; see Methods). An overlapping ‘mask’ stimulus, that did not activate a given neuron when presented alone, selectively inhibits inputs to the neuron that overlap with the mask. As a result, the neuron’s tuning curve was reduced in magnitude and shifted *away* from the mask (Fig 2c, top left panel). This effect is qualitatively similar to contextual shifts in neural tuning curves observed experimentally in cat primary visual cortex (Fig 2c, top right panel) [6].

As a control, we considered a ‘linear-nonlinear’ (LN) model, with responses obtained by a filter followed by a threshold non-linearity: *r*_*i*_ = *f*(∑_*j*_ *v*_*ji*_*s*_*j*_). Linear weights were fitted to match, as closely as possible, the responses of the divisive model across all three stimulus conditions (see Methods). As shown in Fig 2c (lower panel) an LN model was unable to produce the shifts in neural tuning curves observed with the divisive model.

In addition to shifting neural tuning curves, input-targeted divisive inhibition also results in dynamic reshaping of neural receptive fields (RFs). To illustrate this, we extended our previous generative model, to consider the case where presented stimulus features activate sensory inputs, arranged along *two* spatial dimensions. Neural RFs, estimated using reverse correlation with random sparse stimuli (see Methods), exhibited a ‘centre-surround’ structure, with a central excitatory region surrounded by an inhibitory region (Fig 2d, above). However, simultaneously presenting an overlapping grating stimulus dramatically reshaped the estimated RFs, which were elongated orthogonal to the grating (Fig 2d, below). No such contextual shifts in RFs was observed with an LN model (Fig 2e).

Previously, Meister et al. showed that presenting an orientated grating stimulus over a period of several seconds leads to a reshaping of retinal ganglion cell RFs, qualitatively similar to what we observed in our model [17]. (However, note that to properly model the effects of temporal adaption would require extending our work to consider optimal estimation of temporally dynamic stimuli) [18].

**Fig 3:**
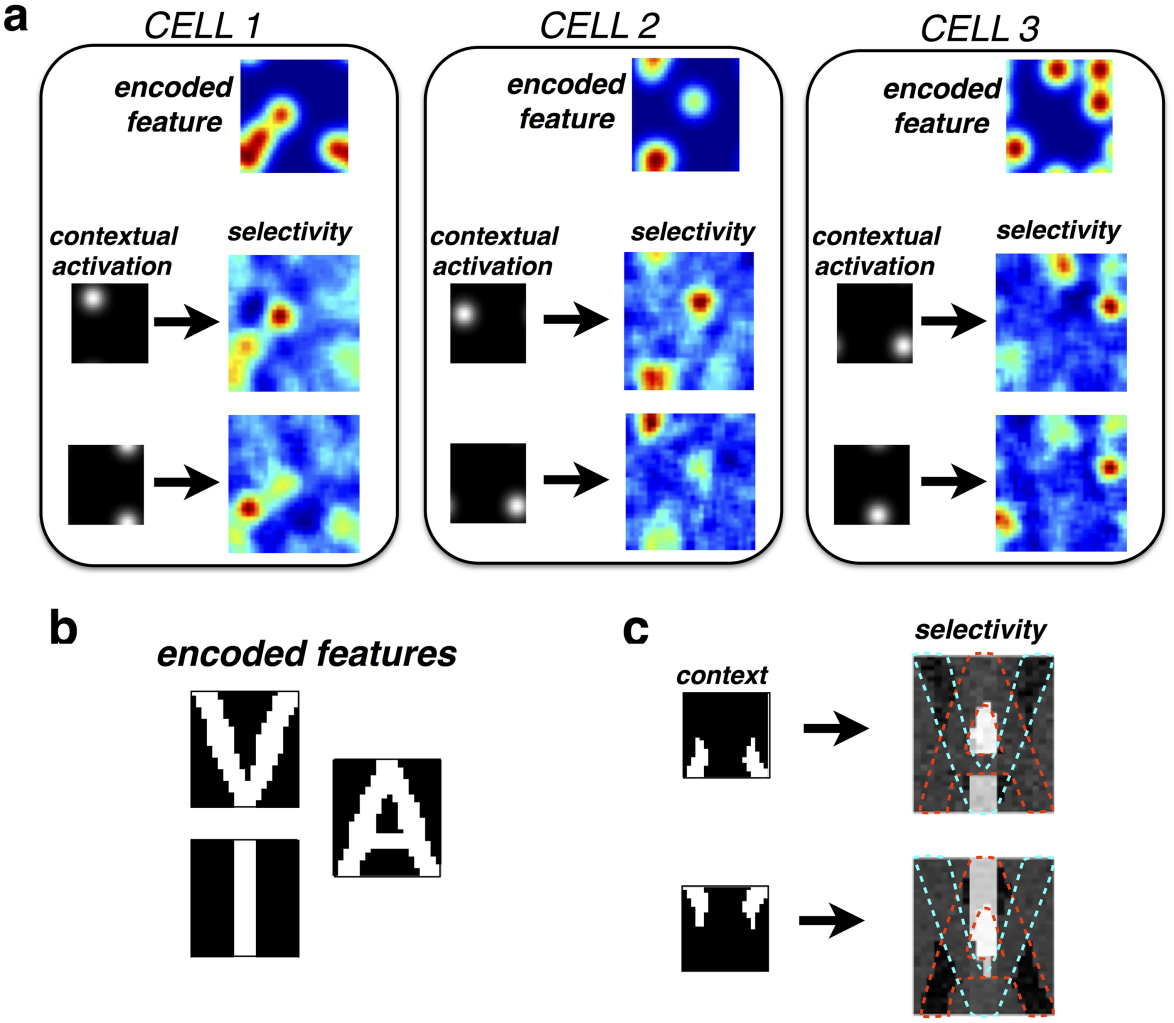
(a) Contextual reshaping of multimodal RFs. Each neuron encodes a stimulus feature (e.g. an odor) that is assumed to elicit a multimodal pattern of sensory activity (upper panels). Neural RFs are measured in the presence of a mask stimulus that activates a small number of nearby receptors. For the three cells shown, recorded RFs undergo complex, non-local changes in the presence of the contextual mask. (b) Reshaping of neural RFs in a simplified network of three neurons, which encode the letters ‘V’, ‘I’, and ‘A’. (c) The RF of a neuron encoding the letter ‘I’ is significantly altered by a contextual stimulus designed to selectively activate one of the other two neurons in the network.

In early visual areas, where neural RFs are localized within a single region of space, our model predicts simple shifts in neural RFs, as shown in Fig 2d. However, in other sensory modalities (e.g. olfaction/audition), where neural RFs have a more complex structure, contextual reshaping of neural RFs could be more complex [19, 20]. To illustrate this, we considered a generative model in which individual sensory features (e.g. presented odors) produce a distributed and multi-modal activation of sensory receptors, as shown in Fig 3a (upper panels; see Methods). We measured the RFs of neurons in response to a random sparse stimulus plus a contextual mask that activated a small subset of nearby receptors. The contextual mask led to complex changes in neural RFs that could not be characterised as a simple repulsive shift away from the context (Fig 3a). Moreover, the observed reshaping of neural RFs was highly non-local: contextual activation of nearby receptors affected distant regions of a cell’s RF.

To explain intuitively this contextual reshaping of neural RFs, we considered a toy generative model consisting of three stimulus features, which produce patterns of sensory activation resembling the letters ‘V’ ‘A’ and ‘I’, respectively (Fig 3b). We measured the RF of the neuron encoding the letter ‘I’ in response to random sparse stimuli, and in the presence of an overlapping contextual stimulus (Fig 3c). Because of the simplicity of this network, we can understand how the contextual stimuli reshape the neuron’s RF. For example, the first contextual stimulus strongly activated the neuron encoding the letter ‘A’ (Fig 3c, top left) leading to targeted inhibition of neural inputs that overlap with the letter ‘A’. As a result, the recorded neuron became insensitive to these inputs, and they did not form part of its recorded RF (Fig 3c, top right). An analogous effect occurred with a contextual stimulus designed to activate the neuron encoding the letter ‘V’ (Fig 3c, lower panels). Note that this contextual reshaping of neural RFs occurred because inhibition was targeted on a subset of neural inputs (Fig 1c); it would not occur in a network with global inhibition, that acted directly on neural responses (Fig 1b).

### Adaptive receptive fields alongside invariant neural code

**Fig 4:**
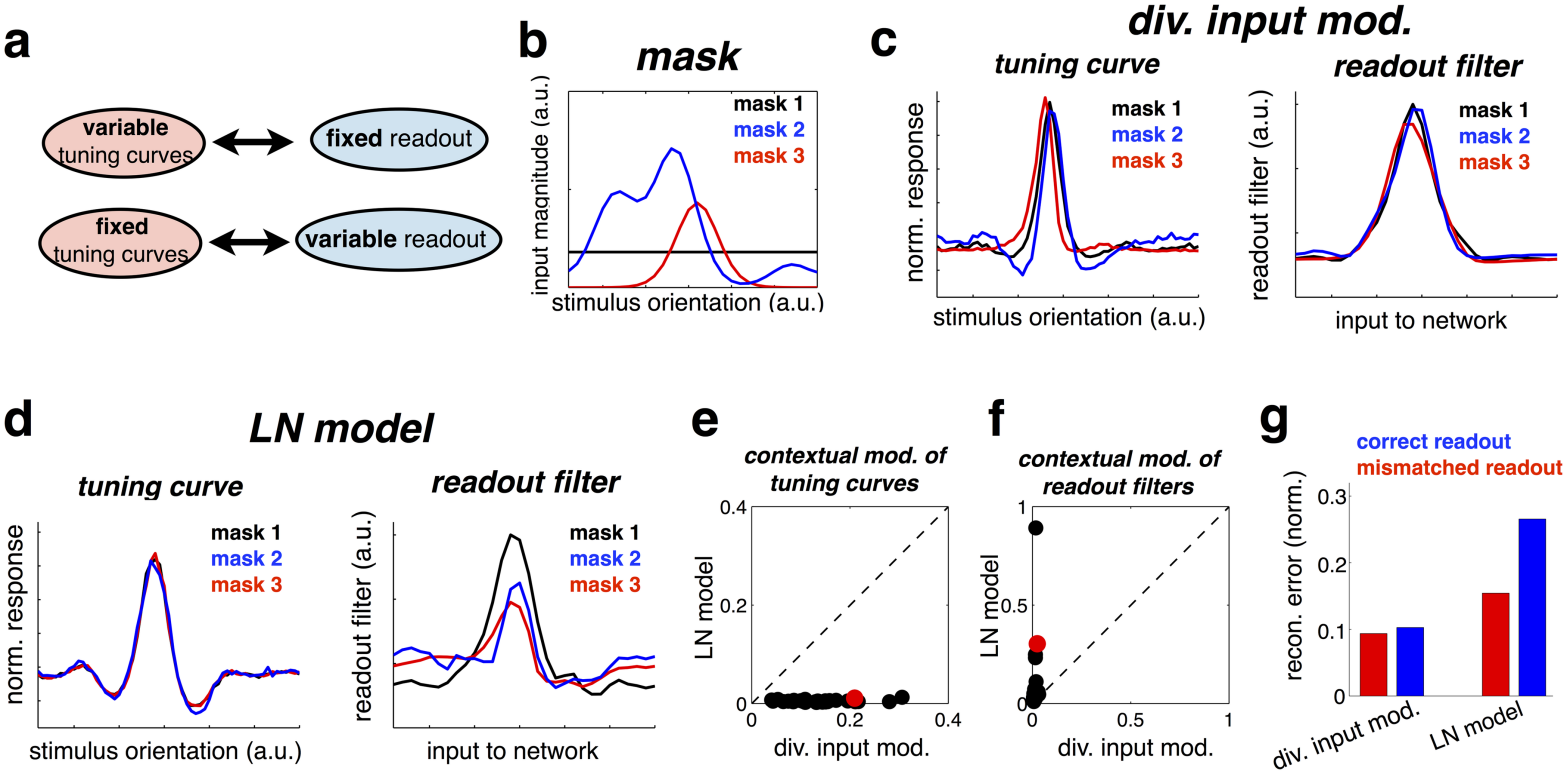
(a) Schematic illustrating how contextual shifts in neural tuning curves required for a context-invariant neural code. (b) Contextual mask presented in each condition. (c, left panel) Tuning curve of a model neuron in the presence of the three different stimulus masks (tuning curves are rescaled, to have zero mean and unitary standard-deviation). (right panel) Inferred readout filters for the same neuron in each condition. (d) As for panel c, but for an LN model. (e) Mean squared difference in (rescaled) tuning curves across the different stimulus contexts. Each cell corresponds to one data point. The example cell, plotted in panels c-d is shown in red. (f) Identical analysis to panel e, but applied to the linear readout filters. (g) Normalized reconstruction error using ‘correct’ readout filters for each stimulus condition (blue bars), or ‘mismatched’ decoders, inferred in other stimulus conditions.

The observation that neurons have highly variable RFs could lead one to conclude that the neural code also varies with stimulus context. However, note that each neuron always encodes a fixed stimulus feature, as defined by the generative weights *w*_*ij*_. As a result, the neural responses can always be read-out in the same way by the downstream neurons, by interpreting the activity of each neuron as indicating the presence of its preferred feature. For this same reason, our model can be extended to hierarchical frameworks where each layer predicts the responses of the layer below (section 3 in S1 Text). The resulting neural code is thus ‘fixed’ (as defined by the features *w*_*ij*_), and the neural representation is ‘invariant’ (in the sense that sensory neurons always represent the same objects, regardless of context). However, in order to maintain this fixed code, neurons in the network need to have variable RFs, that adapt depending on the stimulus context (Fig 4a).

To illustrate this idea, we return to our earlier simulation with bell-shaped tuning curves, shown in Fig 2c. This time, however, we plotted neural tuning curves in the presence of three different ‘contexts’ (Fig 4b; each context was a ‘mask’, constructed from a random combination of ‘background’ stimulus features; these masks were constantly added to the inputs used to measure the tuning curve and estimate the read-out weights). As before, the tuning curves were shifted by the context (Fig 4c, left panel; tuning curves are rescaled and shifted to have the same magnitude and zero mean). Next, we trained ‘readout filters’ to linearly reconstruct the inputs from the neural responses (see Methods). As could be expected, these were similar to the actual read-out weights *w*_*ij*_. In particular, and in sharp contrast with the tuning curves, which were shifted by context, readout filters were almost completely invariant to changes in context (Fig 4c, right panel).

For comparison, we repeated the same procedure with an LN model (Fig 4d). As seen previously, in this model neural tuning curves are not shifted by context (only their gain is changed, which does not appear on the re-scaled tuning curves). However, readout filters *were* altered by context, meaning that in each context, downstream neurons would have to integrate responses from the network differently (depending to the context) in order to reconstruct the stimulus.

As shown in Fig 4e and 4f, the same qualitative effects were observed for the tuning curves and readout filters across the entire neural population, in addition to the example cell shown in Fig 4c and 4d.

Finally, we quantified the reconstruction error across all three conditions (normalized rms error), obtained with the ‘correct’ readout filter (i.e. trained on responses obtained with the same mask; Fig 4g, blue bars), compared with a ‘mismatched’ decoder (trained in different conditions; Fig 4g, red bars). In the input-targeted inhibition model, similar performance was achieved in either case, as the readout filter did not change significantly across conditions. In contrast, in the LN model performance was drastically reduced when using a mismatched decoder, learned in a different context.

Our results suggest that, rather than trying to describe neural responses using a static ‘encoder model’ (e.g. tuning curves or RFs) one may be able to fit a simpler context-invariant ‘decoder model’, describing how to reconstruct the stimulus from neural responses. Experimental support for this is provided by Marre et al. who were able recover a highly accurate reconstruction of a moving bar stimulus from a simple linear readout of retinal ganglion cell responses [21]. In contrast, neural responses in their experiment were poorly described by an LN model.

**Fig 5:**
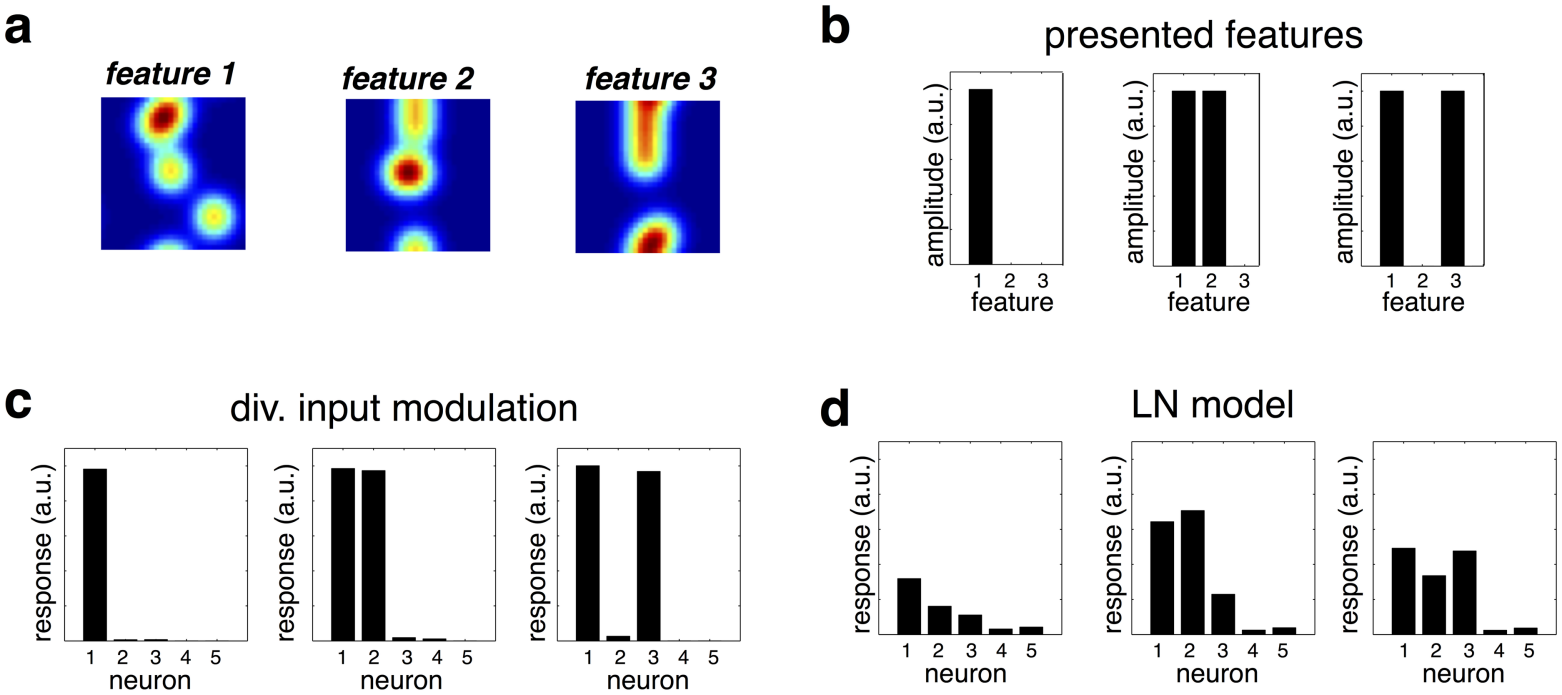
Input-targeted inhibition allows for discrimination of similar stimulus features. (a) Three different stimulus features (e.g. odors) encoded by different neurons in the network. The plots show the overlapping pattern of receptor activation elicited by each feature. (b) Three different combinations of features presented to the network. (c) Neural responses to each feature combination, obtained from the input-targeted divisive inhibition model. The response of each neuron is highly specific to its encoded feature, even with multiple overlapping features presented simultaneously. (d) As for panel c, but with an LN model, trained to match the responses of the divisive input model, to a range of different presented feature combinations. In contrast to before, neurons respond non-specifically when similar featue are presented together.

The advantages of input-targeted divisive inhibition are also seen when discriminating between similar features, presented together. To demonstrate this, we returned to the earlier model with multimodal distributed features, shown in Fig 3a. We considered neural responses to combinations of three similar stimulus features, encoded by different neurons in the network (Fig 5a): feature 1 presented alone, and alongside feature 2 or 3 (Fig 5b). Fig 5c plots the response of five feature-selective neurons. Despite the fact that the three features activated highly overlapping sets of receptors, neural responses were highly specific, with only neurons that encode the presented odors responding on a given trial. In contrast, an LN model could not achieve this degree of specificity (Fig 5d).

### Neural implementation

**Fig 6:**
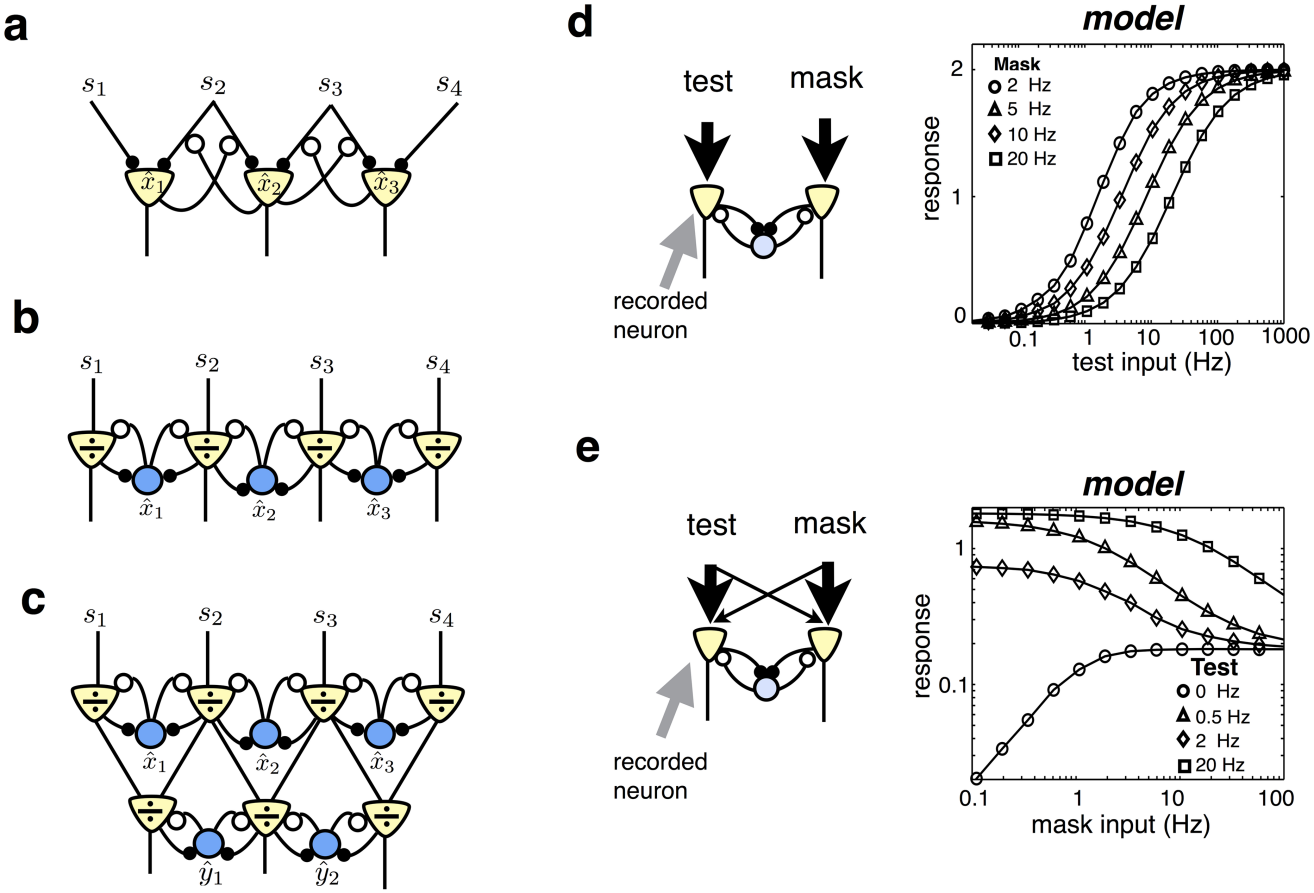
Proposed neural implementation. (a) Example network in which each stimulus feature is encoded by an excitatory neuron that projects to higher level areas. Divisive inhibition acts on individual synaptic inputs. (b) Example network with two neural populations: excitatory neurons encode the ratio between the received and predicted input, $\frac{s_{j}}{\langle s_{j}\;(\hat{x})\rangle }$, while inhibitory neurons encode estimated stimulus features, ${\hat{x}}_{i}$. (c) Example of a hierarchical network. The fractional prediction error encoded in a given layer is integrated by downstream neurons, which encode more complex stimulus features. (d) Divisive gain control. (left) A ‘test’ stimulus activates the input to the recorded neuron (indicated with arrow), while a mask stimulus activates the input to the other neuron. Response of recorded neuron is plotted versus amplitude of the test stimulus. Each plot corresponds to a different amplitude mask (see legend).

The estimation algorithm described by Eq 3 could be implemented in more than one way within a neural network. The most direct implementation would be for each neuron to encode a single stimulus feature, with firing rate proportional to the estimated feature ($r_{i}\propto {\hat{x}}_{i}$). In this case each neuron needs to selectively inhibit the input synapses of neurons encoding different features, as shown in Fig 6a. The response of each neuron evolves in time according to:

$$\frac{\partial r_{i}}{\partial t}\propto \sum_{j}{\tilde{w}}_{ji}\left(r\right)\;s_{j}-\text{const}$$ (4)

where ${\tilde{w}}_{ji}(r)$ is an ‘effective input weight’, obtained by dividing the feed-forward weight, *w*_*ji*_, by the responses of other neurons in the network, according to: ${\tilde{w}}_{ji}\;(r)=\frac{w_{ji}}{\sum_{k}\;w_{jk}r_{k}+w_{0}}$. As a result, feedback connections alter the effective weighting of each input, thereby altering neural stimulus selectivity.

There are two reasons why neural dynamics described by Eq 4 may not be biologically plausible, at least in the cortex. First, the network violates Dale’s law: neurons are required to send both excitatory projection to higher sensory layers and inhibitory feedback to other neurons in the same area. Second, it requires a highly selective form of feedback, targeted on individual synapses (Fig 6a).

To overcome these issues, we propose an alternative network that consists of two distinct neural populations: excitatory neurons that encode the ratio between the received and predicted input, $\frac{s_{j}}{\langle s_{j}\;(\hat{x})\rangle }$, and inhibitory neurons that encode stimulus features, ${\hat{x}}_{i}$ (Fig 6b). Each excitatory neuron receives feed-forward input from one receptor type, and lateral inhibition from interneurons. Its response evolves in time as:

$$a\frac{dr_{j}^{exc}}{dt}=s_{j}-\left(w_{0}+\sum_{k}\;w_{jk}r_{k}^{inh}\right)r_{j}^{exc}.$$ (5)

where *a* is a constant that (along with the magnitude of inhibition) determines the of timescale of excitatory responses.

Inhibition acts multiplicatively on the leak term in the firing rate dynamics (see Discussion for biophysical mechanism). These dynamics ensure that in the steady state the response of each excitatory neuron is equal to the *ratio* of its excitatory and inhibitory input: $r_{j}^{exc}=\frac{s_{j}}{w_{0}+\sum_{k}\;w_{jk}r_{k}^{inh}}$. (Note that, unlike classical subtractive predictive coding, in the case where sensory inputs are perfectly predicted by the network, excitatory responses are equal to unity, not zero).

Inhibitory neurons receive lateral input from nearby excitatory neurons. Their responses evolve in time according to:

$$b\frac{dr_{i}^{inh}}{dt}=\sum_{j}w_{ji}\;\left(r_{j}^{exc}-1\right).$$ (6)

where *b* determines the rate that inhibitory neurons integrate their input. In the steady state (i.e. when $r_{j}=\frac{s_{j}}{w_{0}+\sum_{k}\;w_{jk}r_{k}^{inh}}$), this equation is equivalent to the optimal estimation algorithm shown in Eq 3. Thus, in the steady state, the response of each inhibitory neuron will be proportional to an encoded feature, $r_{i}^{inh}={\hat{x}}_{i}^{opt}$. Both excitatory and inhibitory neural responses are constrained to be positive.

Stimulus features can be recovered by neurons in higher-level areas by temporally integrating the responses of the excitatory neurons (Fig 6c and section 3 in S1 Text). Thus, the network implements a form of ‘predictive coding’, in which the *fractional prediction errors*, rather than the estimated stimulus features themselves, are communicated to higher level sensory areas [1]. In the following sections we will explore the implications of input-targeted divisive inhibition in the context of this ‘predictive coding’ network.

### Sensory gain control

We investigated how divisive inhibition modulates the steady state responses of excitatory neurons, which encode the fractional prediction error. We first considered a very simple model composed of only two sensory receptors, both activated by a single stimulus feature. The corresponding neural network consists of two excitatory neurons that connect with equal strength to one inhibitory neuron (Fig 6d, left).

In this network, the sustained response of each excitatory neuron is simply equal to its feed-forward input, divided by the total rectified input to the network (section 4 in S1 Text):

$$r_{1}^{exc}\propto \frac{s_{1}}{\max(s_{1}+s_{2},w_{0})}.$$ (7)

This equation bears strong similarity to the canonical divisive normalization equation, developed by Heeger et al. [10, 22]. Thus, our normative framework parsimoniously predicts the nonlinearities seen in previous phenomenological models of divisive normalization.

When the feed-forward input to neuron 1 is very weak (i.e. *s*_1_ ≪ *s*_2_), the denominator of Eq 7 is constant, and the neuron’s responses increases linearly with input strength. When the feed-forward input to neuron 1 is very strong (i.e. *s*_1_ ≫ *s*_2_), on the other hand, the numerator and denominator of Eq 7 approach equality, and the neuron’s response saturates. Plotted on a logarithmic scale, this gives rise to a sigmoidal input-response curve (Fig 6d) [2].

Lateral inhibition from a ‘mask’ stimulus that does not provide direct input to neuron 1 (i.e. it activates *s*_2_ only), suppresses the neuron’s response [45]. When *s*_1_ ≫ *w*_0_, the effect of the mask is to add an additional constant to the denominator of Eq 7, shifting the neuron’s input-response curve to the right on a logarithmic scale (Fig 6d). Consequently, a stronger feed-forward input is required to elicit the same neural response.

A mask stimulus that provides weak input to neuron 1 and strong input to neuron 2 (i.e. it weakly activates *s*_1_, and strongly activates *s*_2_, as shown on Fig 6e) can both suppress or facilitate the response of neuron 1, depending on the strength of the neuron’s feed-forward input [2]. When the feed-forward input to neuron 1 is very weak, the denominator of Eq 8 is constant (due to rectification), and the neuron linearly sums its feed-forward inputs. As a result, its response is facilitated by the mask (Fig 6e). When the feed-forward input to neuron 1 is strong, the mask increases the size of the denominator, suppressing the neuron’s response (Fig 6e).

The results described above also apply to larger networks consisting of many excitatory and inhibitory neurons. Indeed, for “simple” inputs that do not activate multiple overlapping feature detectors, the sustained response of each excitatory neuron is approximately equal to its feed-forward input, divided by the summed input to nearby neurons (Section 4 in S1 Text):

$$r_{i}^{exc}\propto \frac{s_{i}}{\max(\sum_{j}\;w_{ij}s_{j},w_{0})}.$$ (8)

Thus, the classical normalization model [10], that was originally designed to provide a phenomenological description of non-linearities in neural responses, emerges as a special case of our proposed dynamics.

### Temporal dynamics of neural responses

**Fig 7:**
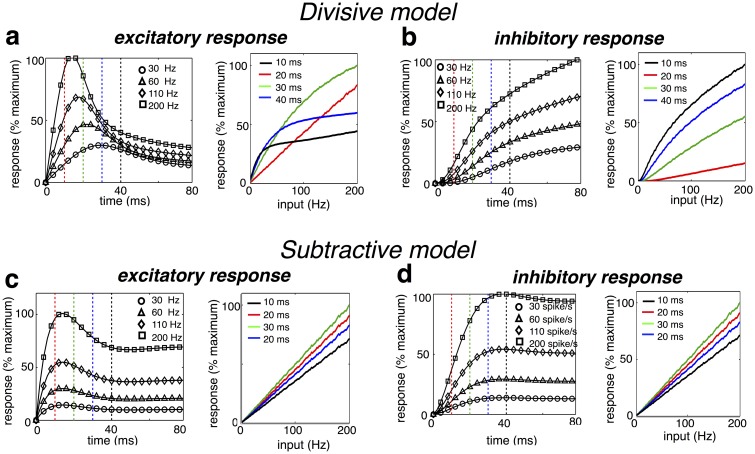
Predicted temporal response profile. (a, left) Temporal response profile of excitatory neuron, to a constant feed-forward input of varying strength. (right) Instantaneous response of the excitatory neuron versus amplitude of feed-forward input. Each plot corresponds to a fixed time after stimulus presentation (indicated by vertical dashed lines in left panel). (b) Same as (a), but for an inhibitory model neuron. (c-d) Same as a-b, but for a model with subtractive, rather than divisive inhibition.

We next investigated the temporal dynamics of excitatory and inhibitory neural responses to a constant stimulus in the simple, two neuron network described in the previous section (Fig 6d, left panel). Following stimulus onset, the response of the activated excitatory neuron, encoding the fractional error signal, exhibited a transient peak in activity followed by a decay (Fig 7a). At the same time, the response of the inhibitory neuron, which encoded the sensory estimate, increased continuously towards the steady state (Fig 7b). This qualitative behaviour is a general property of predictive coding, and thus also occurred for the subtractive model, where excitatory neurons encoded the absolute (rather than the fractional) error (Fig 7c and 7d).

What distinguishes the subtractive and divisive models was the input-depencence of the neural dynamics. For the divisive model, the timescale of excitatory neural responses decreased with the sensory input, resulting in a shorter time to peak response with higher amplitude inputs (Fig 7a). This is because the leak term in the excitatory neural dynamics (which implements divisive inhibition) is proportional to its inhibitory input Eq (5). Thus, the greater a neuron’s inhibitory input, the quicker its response varies in time. In contrast, the temporal dynamics of the subtractive model were input-invariant.

Recent experiments using voltage sensitive dye to measure V1 responses reported contrast-dependent temporal dynamics, consistent with our model [23]. Similarly, Albrecht et al. [24] observed that the time to peak firing rate response decreases with visual contrast. However, Albrecht et al also reported that temporally shifting firing rate responses to compensate for contrast-dependent variations in onset latency resulted in temporal response profiles that were approximately contrast invariant. This discrepancy between voltage data and firing rate data could be accounted for by including a firing threshold into our model.

**Fig 8:**
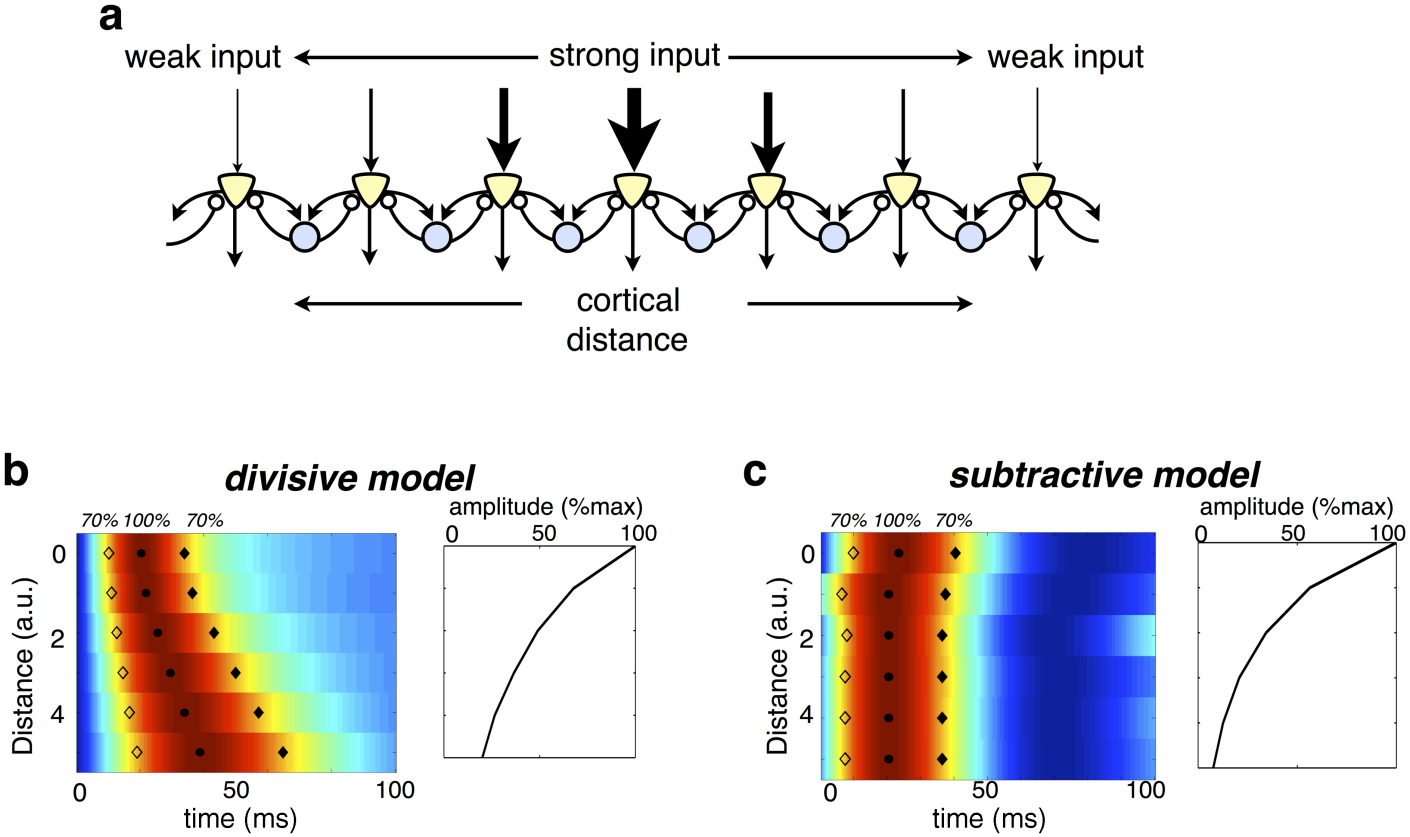
Traveling waves in the visual cortex. (a) Schematic of topographic model network, in which each inhibitory neuron connects with equal strength to two neighbouring excitatory neurons. The feed-forward input decreases with distance from the centre. (b) Heat map of excitatory neural responses, normalized by peak amplitude. Each row shows the response of a neuron at a specified distance from the centre. Filled and solid diamonds indicate at what time each neuron’s response is 70% of its maximum. The right panel indicates the maximum response of each neuron. (c) Same as c, but with subtractive, rather than divisive inhibition.

When put into the context of a larger, topographically organized sensory layer, the temporal dynamics of the divisive model could parsimoniously account for the presence of ‘traveling waves’ observed in the visual cortex, where a presented stimulus generates a wave of activity that spreads gradually outwards from a single cortical location (Fig 8a) [25]. According to our model, traveling waves will occur when the input generated by a stimulus varies in strength with cortical location [26] (Fig 8b). Neurons that receive strongest feed-forward input will respond quickest, followed by nearby neurons that receive weaker input. The resultant effect is a damped traveling wave that spreads outwards from neurons most strongly activated by the stimulus (Fig 8c). In contrast, with subtractive inhibition, the timecourse of neural responses does not depend on their distance from the input (Fig 8d).

## Discussion

### Functional role of divisive inhibition

It has long been thought that divisive inhibition performs a kind of gain control, that keeps neural firing rates within their available dynamic range [10, 27, 28]. Here we provide an alternative interpretation, that divisive inhibition occurs as a consequence of optimal cue combination given sensory noise. When the variance of each input depends on its mean, some signals become more reliable than others. Divisive inhibition insures that each signal is weighted appropriately, before these signals are combined by downstream neurons.

In that sense, our work places itself in the more general framework of optimal cue combination [29] where each cue should be weighted according to its reliability before being combined. Human subjects are indeed able to perform such optimal cue combination [30, 31], and are also able to implement explaining away, e.g. to resolve ambiguities by assigning inputs to their most likely sources [32]. Our model proposes that optimal cue combination and explaining away are already implemented at a microscopic level by sensory networks, through selective divisive gain modulation of sensory neural responses.

In contrast to the gain-control hypothesis, our framework precisely specifies the form of divisive inhibition required for optimal estimation, which should occur *before* individual inputs are combined (Fig 1c). For simple stimuli, which activate only one feature detector at a time, the predicted neural responses are consistent with the classical divisive normalization model (Figs 6 and 7). However, for more complex stimuli, which activate multiple overlapping feature detectors, input-targeted divisive inhibition results in dynamic changes in neural tuning properties and receptive field shapes (Figs 2 and 3), not captured by the classical divisive normalization model.

A prediction of our model is that sensory normalisation will vary with changes in neural variability. Thus, future experimental tests of our work could investigate whether divisive normalisation is altered as expected by stimulus-dependent modulations in neural Fano-factor (see section 1.2 in S1 Text) and noise correlations (see section 2 in S1 Text) [33, 34].

### Comparison with other functional accounts of divisive inhibition

Previously, Schwartz and Simoncelli showed that divisive normalisation can serve to remove statistical redundancies between neural responses, leading to a more efficient code [35]. In a later extension to this work, they showed that divisive normalisation can be interpreted as implementing ‘explaining away’ of global stimulus features (e.g. global image contrast) so as to permit optimal inference of local stimulus features (e.g local reflectance) [36].

While in both our model and that of Schwartz et al., divisive normalisation implements explaining away, their underlying assumptions are very different. In Schwartz et al.’s model, normalisation is predicted because of the assumed high-level structure of sensory signals, as being produced by multiplying local and global stimulus features. In contrast, in our model, divisive normalisation is predicted due to the biophysics of sensory signal transduction, which leads to sensory signals being corrupted by signal-dependent (and not Gaussian) noise.

Schwartz & Simoncelli’s model also belongs to a broader class of normalisation models in which divisive occurs after sensory inputs have been combined [2]. In contrast, our model predicts that divisive normalisation should act directly on the inputs, before combination. As we showed (Fig 2), such pre-combination divisive inhibition leads to flexible RFs, which are dynamically shifted by the stimulus context. In contrast, output-targetted divisive normalisation will only lead to such shifts in neural RFs when sensory inputs undergo an additional (e.g. quadratic) non-linearity before normalisation.

Previously Beck et al. proposed a new role for divisive normalisation in performing a probabilistic compuation known as ‘marginilisation’ [37, 38]. This computation is required for many different tasks, in which one wants to infer a subset of ‘relevant’ stimulus features, while disregarding (i.e. marginilising) other irrelevant features. At some level, this explanation is related to Schwartz et al.’s work, where normalisation was assumed to factor out (i.e. marginilise) global fluctuations in the sensory input, so as to allow inferences about local features. However, Beck et al.’s model differs from both Schwartz et al. and our work, in that marginilisation is predicted as a result of a particular type of probabilistic neural population code.

In previous work, we proposed a model in which input-targeted divisive inhibition implements competition between different stimulus features. However, this model relied on a number of assumptions about sensory stimuli (e.g. that they were produced by binary stimulus features that had Markov temporal dynamics), as well as assumptions about the spiking neural code [18]. Here we show that input-targeted divisive inhibition emerges very generally, and irrespective of additional assumptions about the neural code and signal dynamics, so long as the sensory noise scales with the magnitude of the signal.

Recent experimental work suggested that in the ferret auditory cortex, neural responses adapt to the stimulus statistics in such a way as to allow behaviourally relevant signals to be extracted from background noise [39, 40]. Interestingly, Mesgarini et al. showed that their results could be explained by top-down divisive feed-back. While the details of our model differ from that of Mesgarini et al. (e.g. they assumed that divisive inhibition acts after inputs are combined) it gives a suggestion as to why top-down divisive feed-back could result in the noise-invariant neural responses observed in their data.

### Divisive versus subtractive predictive coding

Predictive coding implies that, rather than directly encoding sensory stimuli, feed-forward neurons encode a prediction error that can be used to update the internal representation in higher-level areas [1]. Here we show that, given signal-dependent sensory noise, this error signal should take a fractional form, implying divisive inhibition.

Previously, Spratling et al. showed that a predictive coding network that minimizes fractional prediction errors can account for a number of classical and extra-classical response properties of neurons in visual area V1 [41]. We provide a normative interpretation of Spratling’s model, as implementing optimal estimation of presented stimulus features given signal-dependent noise. We find that, for a large family of distributions in which the variance in each input is proportional to its mean (including, but not limited to Poisson noise), the prediction errors take a fractional form, implying divisive predictive coding (see section 1 in S1 Text).

With the exception of Spratling’s work, previous predictive coding models have usually assumed that sensory neurons encode the *difference* between their received and predicted input [1, 42]. This type of code will be optimal only if the the variance in each sensory input is constant, irrespective of its mean. Subtractive predictive coding results in qualitatively different neural response properties, compared to divisive predictive coding. It predicts that: (i) the time course of neural responses is independent of stimulus strength; (ii) neural responses vary linearly with their feed-forward input, and thus, do not saturate; (iii) neural RFs are largely invariant to changes in stimulus context (see section 4 in S1 Text). In summary, subtractive predictive coding cannot account for many of the non-linear response properties observed in sensory neurons, and that are explained by divisive predictive coding.

### The sources of neural noise

Here, we show that the optimal form of neural gain control depends on how neural inputs are corrupted by noise. Specifically, signal-dependent noise requires input-targeted divisive inhibition, in contrast to gaussian noise, which requires global subtractive inhibition.

‘Noise’ here refers to the trial-by-trial variability of neural inputs, given a fixed stimulus. Generally, multiple noise sources combine to produce neural variability, including external noise sources (e.g. random fluctuations in light intensity), and internal noise sources (e.g. spike failure). The model is agnostic to these details, as long as the trial-by-trial variability of inputs to the network scales monotonically with their amplitude.

In contrast, the model network itself has deterministic dynamics: for a given input, the neural responses are always be the same. However, while this choice was made for simplicity, related work on networks of spiking neurons shows how optimal estimation can be performed in a network of neurons that exhibit Poisson-like spiking statistics [18, 43]. In these models, internal noise fluctuations that alter the spike times of single neurons are compensated by recurrent connections in the network, such that the read-out from the population response is relatively stable.

### Circuits and mechanisms underlying divisive inhibition

The effects presented here come about as a result of optimal estimation with signal-dependent noise, and are thus largely independent of the specific neural mechanism that implements divisive inhibition. For example, contextual reshaping of neural RFs (Figs 2 and 3) occurs because ‘explaining away’ takes place at the level of the inputs, before they have been combined, while gain modulation of neural responses (Fig 6) is a property of the fractional prediction error.

Nonetheless, in order to make concrete predictions about sensory neural responses we proposed a simple network architecture, in which excitatory neurons encode a fractional prediction error, and receive lateral inhibition from local interneurons that encode individual stimulus features (Fig 6b). However, note that there is more than one way to implement the optimal estimation described by Eq 3. For example, divisive inhibition could be mediated via top-down feed-back from higher-level areas [1], or via lateral inhibition of individual synaptic inputs [18] (Fig 6a). However, as they share the same normative structure to our model, these different network architectures result in very similar predictions for the neural responses.

In our proposed network, excitatory neurons at the first level of processing each receive input from one type of receptor, and divisive inhibition from lateral interneurons. This closely matches the observed anatomy of both fly and mouse olfactory system, where mitral cells (or 2nd-order PNs in fly) receive feed-forward input from one type of olfactory receptor, and lateral inhibitory feed-back that depends on the responses of many receptors [42, 44]. Furthermore, recent experiments have shown that in the fly, inhibition from lateral neurons is well described by the exact same divisive formula as obtained with our model [45].

Recently, researchers have reported how various interneuron types play different roles in sharpening and/or globally suppressing visual neural responses [46–49]. While generally, our simplified model is not designed to address this level of detail, it is worth noting that when implemented in a hierarchy (Fig 6c), interneurons at different levels of processing will have qualitatively different effects on the tuning curves of excitatory neurons. Specifically, interneurons in the previous layer to a recorded neuron, that target its *inputs*, will act to sharpen and reshape the neuron’s selectivity, whereas interneurons in the same layer, that provide direct lateral inhibition, will lead to a global suppression (but no sharpening) of its responses.

In our model, divisive inhibition is implemented via lateral feedback from inhibitory interneurons, which multiplicatively increases the ‘leak’ term in the dynamics of the excitatory neural responses Eq (5). A potential candidate for this gain modulation is shunting inhibition [2] (although see [50–52]). More generally however, current experiments suggest that there is not one unique neural mechanism that implements divisive inhibition [22]. Rather a host of different mechanisms, such as synaptic depression [53], ongoing network dynamics [54], and neuromodulatory feedback [55] may be responsible for divisive inhibition in different sensory areas and species. This is consistent with our framework, which suggests that it is the computation performed by divisive inhibition, rather than its neural implementation, that is conserved across sensory systems in order to optimally infer the state of the environment.

### Predicted differences between excitatory and inhibitory responses

The proposed neural network predicts several qualitative differences between the responses of excitatory neurons, which encode fractional prediction errors, and inhibitory neurons, which encode stimulus features. These differences are: (i) long-range (i.e. between cortical regions) signals are normalized, while short-range (i.e. within region) signals are not; (ii) inhibitory neurons respond to more complex non-local features than excitatory neurons in the same area (they are thus expected to exhibit wider, apparently less selective tuning curves, as indeed observed experimentally [56]); (iii) inhibitory responses are less transient than excitatory neural responses.

Recent experiments, using optogenetic techniques, have shown that parvalbumin (PV)-expressing inhibitory cells can have a divisive effect on excitatory responses to sensory stimuli. Interestingly, PV cells appear to fulfil many qualitative criteria required by inhibitory cells in our model, such as broad stimulus tuning, temporally sustained responses, and minimal contrast normalisation (relative to layer 2/3 excitatory neurons, to which they provide input) [48]. Future research will be required to quantify more precisely how the activity of PV cells compares to the predictions of our model.

### The role of feedback

Our model can easily be extended to consider sensory processing in a hierarchy, with neurons at each layer of the network reconstructing stimulus features of increasing complexity based on the inputs they receive from the previous layer (see Fig 6c and section 3 in S1 Text). In this case, optimal estimation also requires using high-level knowledge to constrain and shape the low-level sensory representation. This can be easily incorporated into our framework, in the form of top-down feedback. As well as carrying information about the stimulus features encoded by higher-level areas, this top-down feed-back could also carry information about the organism’s prior experience and task-demands. Future work could investigate whether such top-down feedback is able to account for the experience-dependent and attention-dependent shifts in neural tuning curves that are observed experimentally [57, 58].

In summary, our model suggests a highly dynamic system, in which neural RFs and tuning curves are continuously reshaped by the spatiotemporal context of presented stimuli, as well as the organism’s prior experience and task-demands. However, the neural code is context invariant: neurons always represent the same external feature, and thus their response can be read the same way by downstream neurons, regardless of the stimulus context.

## Materials and methods

### Subtractive model

In addition to the model described in the main text, we also considered an artificial example, where the input signal is corrupted by constant Gaussian noise (whose magnitude is independent of the signal strength). In this case, encoded features vary in time according to:

$$\frac{\partial {\hat{x}}_{i}}{\partial t}=\eta \sum_{j}w_{ji}\;\left(s_{j}-\sum_{k}\;w_{jk}{\hat{x}}_{k}+w_{0}\right)$$ (9)

Thus, the estimate of each feature evolves in time according to a sum of ‘absolute prediction errors’, $s_{j}-\langle s_{j}\;(\hat{x})\rangle$, equal to the difference between received and predicted inputs.

Note that because of the linearity of this equation, the left hand-side can be rewritten as the sum of a feed-forward input term ∑_*j*_ *w*_*ji*_*s*_*j*_ and a lateral subtractive inhibition term $-\sum_{jk}\;w_{ji}w_{jk}{\hat{x}}_{k}-w_{0}$. In the particular case of constant gaussian noise, lateral inhibition is thus separable and can be seen as occurring “after combination” of these input signals. Similarly, in the steady state, the estimated features can be obtained by a weighted linear sum of feed-forward inputs: ${\hat{x}}_{i}=\sum_{j}\;v_{ji}s_{j}$, with feed-forward weights ***v***_*i*_ directly related to the encoded features ***w***_*i*_ (i.e. $v_{i}={\left(W^{T}W\right)}^{-1}w_{i}$). In that interpretation, competition between encoded features adds a subtractive component (an inhibitory surround) to a static feed-forward filter (Fig 1b).

### Comparison between subtractive and divisive models

For the initial simulations shown in Figs 2–5, we sought to investigate the general implications of divisive versus subtractive inhibition Eqs (3) and (9), irrespective of the specific neural implementation. Although we assumed that neurons encode individual stimulus features, with firing rate proportional to the encoded feature ($r_{i}\propto {\hat{x}}_{i}$), the qualitative results would also be the same for a distributed code, in which each neurons encode a linear combination of stimulus features, according to, $r_{i}\propto \sum q_{ki}{\hat{x}}_{i}$. Note that for the simulations used to generate Figs 2–5 the dimensions are essentially arbitrary, and thus all parameters are quoted in unit-less dimensions. Encoded features were initialized at zero, and updated using Eq 3 for the divisive algorithm, and Eq 9 for the subtractive algorithm. The update rate, *η*, was set to ensure smooth dynamics, while the number of iterations, *N*, was chosen to allow the estimates to converge on steady state values. The background rate, *w*_0_, was set to 0.01. In our framework, the generative model describing how external stimulus features activate sensory receptors determines the network connectivity. Furthermore, in the case where each neuron encodes a separate stimulus feature, there is a one-to-one correspondence between the structure of the generative model (parameterized by ***w***) and the feed-forward connectivity in the network. Specifically, the parameter *w*_*ji*_, that determines how strongly the *i*^*th*^ feature activates the *j*^*th*^ receptor, also determines the connection strength between the *i*^*th*^ neuron and the *j*^*th*^ receptor.

### Comparison between input-targeted divisive inhibition and LN models

We compared the input-targetted divisive inhibition model to a linear-nonlinear (LN) model, with responses obtained by linearly filtering the sensory inputs then applying a static non-linearity: *r*_*i*_ = *f*(∑_*j*_ *v*_*ji*_*s*_*j*_ + *v*_0_). (Note that this is a simple generalisation of the subtractive model where responses were strictly linear). For our simulations we used a threshold non-linearity, while linear weights (*v*_*ji*_) and offset (*v*_0_), were learned so as to best fit the responses obtained with the input-targetted divisive model. Using a different non-linearity (e.g. exponential) had no qualitative effect on the predicted contextual tuning curve changes. In addition, we also considered a ‘global divisive-inhibition’ model (Section 5 in S1 Text).

### Suppression by a contextual stimulus

For the simulation shown in Fig 2b there were 30 sensory receptors and 30 neurons. We used a generative model in which each feature activates two neighbouring receptors (i.e. *w*_*ii*_ = *w*_(*i*+1)*i*_ = 40). Thus, each neuron received equal strength feed-forward inputs from two neighbouring receptors (Fig 2a). We computed the steady-state response of the *k*^*th*^ neuron with both the subtractive or divisive algorithms, in three different stimulus conditions. In the ‘no-context’ condition, only one of the inputs to the recorded neuron was active, with firing rate drawn from a Poisson distribution with mean 50 (i.e. 〈*s*_*k*+1_〉 = 50). For the ‘adjoint context’ condition, a neighbouring input that did not drive the recorded neuron was also active (with amplitude 〈*s*_*k*+2_〉 = 20). Finally, for the ‘disjoint context’ condition, an input on the opposite side of the recorded neuron’s receptive field was active (i.e. 〈*s*_*k*−1_〉 = 20). In each condition, we averaged the neuron’s steady state response over 200 trials.

### Contextual shifts in tuning curves

For the simulation shown in Fig 2c there were 30 sensory receptors and 30 neurons. We assumed a generative model in which a stimulus moving in a given direction (indexed by ‘*i*’) activates multiple neighbouring receptors, described mathematically via the circular basis functions: $w_{ji}=w_{max}e^{4[\cos(\frac{2\pi }{30}(j-i))-1]}$ (with *w*_*max*_ = 40). As before, this implies that each neuron receives feed-forward inputs from multiple neighbouring inputs. We first looked at the steady state response of a single neuron to a varying stimulus direction, *i*. The activation of the *j*^*th*^ sensory input was drawn from a Poisson distribution, with mean 〈*s*_*j*_ (*i*)〉 = *w*_*ji*_ + *w*_0_. We next looked at the response of the same neuron in the presence of a ‘mask’, which activated a single receptor, shifted 3 receptors to the left or right of the neuron’s preferred input. The activation of this receptor was held constant at 200. The input to all other receptors was the same as in the previous control condition. The mask was chosen specifically so that it did not elicit any response in the recorded neuron when presented alone.

### Measurement of receptive fields

For the simulation shown in Fig 2d and 2e there were 400 neurons, and 900 sensory inputs (arranged on a 30×30 grid in visual space). Each neuron encoded a circular ‘blob-like’ stimulus feature. Specifically, columns of the matrix *W* specified the feature encoded by each neuron, with elements given by: $w_{ji}=w_{max}e^{-\frac{1}{2\sigma_{w}^{2}}[{\left(x_{j}-x0_{i}\right)}^{2}+{\left(y_{j}-y0_{i}\right)}^{2}]}$. *x*0_*i*_ and *y*0_*i*_ specify the preferred region of visual space for the *i*^*th*^ neuron, distributed uniformly along the axis spanned by *x* and *y* (0 → 1). *w*_*max*_, and *σ*_*w*_ determine the amplitude and width of the encoded features, and were set to 40 and 0.1 respectively. We first performed a simulation with ‘random sparse’ stimuli. Sensory inputs, *s*_*j*_, were either equal to 0 (with probability 0.95) or 100 (with probability 0.05). Next, a vertical grating stimulus (in which each bar spanned 8 pixels), of magnitude 20, was added to the random sparse stimulus (Fig 2d and 2e, bottom left). The phase (but not the orientation) of the grating varied randomly on each trial. Thus, on the *n*^*th*^ trial, the sensory input was equal to, $s^{n}=s_{grating}^{n}+s_{noise}^{n}$. In each case, neural receptive fields (RFs) were quantified using reverse correlation: ${\hat{w}}_{j}=Q_{ss}^{-1}q_{rs}^{j}$, where (*Q*_*ss*_)_*ij*_ = 〈*s*_*i*_*s*_*j*_〉 and ${\left(q_{rs}\right)}_{i}^{j}=\langle s_{i}r_{j}\rangle$, and 〈⋅〉 denotes an average over 10^4^ stimulus presentations.

In Figs 3a and 5 we considered an ‘olfactory network’, with neurons were assumed to have a distributed selectivity, spanning multiple receptor inputs. Mathematically, the network was similar to the network described above. However, for the olfactory simulations, the feature encoded by each neuron consisted of a sum of four ‘blobs’, distributed randomly across the input space (see examples shown in upper panels of Fig 3a).

Neural receptive fields were estimated as before, in response to a random sparse stimulus plus a contextual stimulus. For the plots shown in Fig 3a, the contextual stimulus consisted of a single ‘blob’ (of magnitude 100, and width *σ*_*cntxt*_ = 0.1), that activated a set of nearby receptors (see black and white panels in Fig 3a).

Finally, we illustrated the principles underlying reshaping of neural receptive field using a simple network of only three neurons, each of which encoded a different letter of the alphabet (‘A’, ‘I’, and ‘V’). Encoded features (comprised of 600 sensory inputs, arranged in a 20×30 grid), are shown in Fig 3b. As before, neural RFs were estimated using random sparse stimuli, in addition to a contextual mask (shown in the left panels of Fig 3c).

### Invariance of neural code

We next investigated how divisive inhibition enables the network to maintain an invariant representation of encoded stimulus features.

The network used for these simulations was the same as the model with bell-shaped tuning curves, shown in Fig 2c. We measured tuning curves in the same way as before, measuring the mean firing rate of each neuron versus the stimulus orientation. However, in this case we measured tuning curves in the presence of three constant ‘masks’, constructed from different combinations of encoded features (Fig 4b), added to the varying stimulus.

In each stimulus condition, we estimated the linear filters required to reconstruct the stimulus from the neural responses, using linear regression. Thus, readout filters were given by $U=\langle \bar{s}{\bar{r}}^{T}\rangle \;{\langle \bar{r}{\bar{r}}^{T}\rangle }^{-1}$, where $\bar{s}=s-\langle s\rangle$ and $\bar{r}=r-\langle r\rangle$.

Fig 4d was constructed in the same way using the LN model (Fig 4e). The parameters of the LN model were fitted to minimize the mean squared difference between the responses predicted by the LN model and the input-targeted inhibition model, across all three stimulus conditions.

In Fig 5 we demonstrate how a model with input-targeted inhibition is able to discriminate between similar overlapping stimulus features. To illustrate this, we returned to the ‘olfactory network’ used to generate Fig 3a. We compared this input-targeted divisive inhibition model to the output-targeted divisive inhibition model, described previously. Parameters of this model were fitted to minimize the mean squared difference between the responses of the global inhibition model and the input-targeted inhibition models. Stimuli used to fit the model parameters consisted of random linear combinations of the features encoded by the network, corrupted by Poisson noise.

### Neural network implementation

We proposed a neurally plausible implementation of the the estimation algorithm described in Eq 3 (Fig 6b). This network consists of two populations of neurons: excitatory neurons with dynamics described by Eq 5, and inhibitory neurons with dynamics described by Eq 6. Figs 7 and 8 were generated using discretized version of these equations. For these simulations, the background input was set to *w*_0_ = 1. Parameters, *a* and *b*, determining the timescale of excitation and inhibition were set to 0.08 and 40 respectively (see section 6 in S1 Text for the effect of varying the excitatory and inhibitory timescales). Input spikes were always counted over a time-window of *T* = 1s, so that the number of spikes fired by each input is equal to its firing rate.

The network connectivity was entirely constrained by the generative model describing how presented stimulus features activate the inputs to the network. That is, the parameter ‘*w*_*ji*_’, that describes how strongly the *i*^*th*^ stimulus feature activates the *j*^*th*^ receptor, also determined the strength of the lateral connection between the *j*^*th*^ excitatory neuron and the *i*^*th*^ inhibitory neuron.

### Gain modulation

For the plots shown in Fig 6d and 6e we considered a minimal model with 1 encoded feature and 2 sensory inputs. Within our framework, this corresponds to a network with 1 inhibitory and 2 excitatory neurons (Fig 6d, left panel). The inhibitory neuron received equal strength inputs from both excitatory neurons (*w*_11_ = *w*_21_ = 40). Steady-state excitatory responses could be obtained directly from Eq 7. In Fig 6d, the input to each excitatory neuron was drawn from a Poisson distribution with mean: 〈*s*_1_〉 = *I*_*test*_ and 〈*s*_2_〉 = *I*_*mask*_ respectively. in Fig 6e, the ‘test’ and ‘mask’ stimulus activated both sensory inputs, so that: 〈*s*_1_〉 = *I*_*test*_ + 0.1*I*_*mask*_ and 〈*s*_2_〉 = *I*_*mask*_ + 0.1*I*_*test*_.

### Temporal response dynamics

For the plots shown in Fig 7 we again considered the minimal network with 1 excitatory and 2 inhibitory neurons (connection strengths were same as for Fig 6). On each trial, the input to the recorded excitatory neuron was drawn from a Poisson distribution, with mean varying between 0 & 200Hz. The other neuron received zero input.

For the plots shown in Fig 8, we considered a ‘topographic’ network of 30 excitatory and 30 inhibitory neurons. Each inhibitory neuron connected with equal strength to 2 neighbouring excitatory neurons (*w*_*ii*_ = *w*_*i*(*i*+1)_ = 40). The input to the *j*^*th*^ excitatory neuron was drawn from a Poisson distribution with mean, $\langle s_{j}\rangle =150e^{-\frac{\left|j-k\right|}{2}}$, where *k* denotes the neuron that receives maximal input.

## Supporting information

S1 Text Supplementary appendix. Includes derivation of optimal estimation algorithm, generalisation to non-poisson noise statistics, correlated input noise, and implementation of in a multi-layer neural network.
(PDF)

S1 Fig Comparison between simulation and analytic results. The network consists of 30 inhibitory and 30 excitatory neurons. Connection strengths between inhibitory and excitatory neurons are chosen from a uniform distribution between 0 and 40. In the ‘no mask’ condition one of the sensory inputs varies between 10^−1^Hz and 10^4^Hz, while all the other inputs are set to the background input of *w*_0_ = 1Hz. In the ‘mask condition’ all sensory inputs are activated at 10Hz. Solid lines plot the steady-state response of the maximally driven excitatory neuron, in the ‘no mask’ and ‘mask’ conditions. Dashed curves show the response of the neuron, approximated using eq 31 in S1 Text.
(TIF)

S2 Fig Predictions of global divisive inhibition. The figure is the same as Fig 2c and 2d and in the main text, but with a global divisive inhibition (with responses described by eq 34 in S1 Text).
(TIF)

S3 Fig Effect of varying exicitatory/inhibitory timescales. The figure is the same to Fig 7a and 7b in the main text, with the exception that the timescale of inhibition has been increased by a factor of 40, relative to excitation.
(TIF)
