# Supplementary material for: Sensory noise predicts divisive reshaping of receptive fields
Source: PLoS Comput Biol. 2017 Jun 16;13(6):e1005582. doi: 10.1371/journal.pcbi.1005582 (PMC5509365; doi:10.1371/journal.pcbi.1005582)
Supplement: S1 Text — Includes derivation of optimal estimation algorithm, generalisation to non-poisson noise statistics, correlated input noise, and implementation of in a multi-layer neural network. (PDF) [file pcbi.1005582.s001.pdf]

Sensory noise predicts divisive reshaping of receptive fields Supplementary text

Matthew Chalk, Paul Masset, Boris Gutkin, Sophie Deneve

1 Optimal estimation with signal-dependent noise

Consider the situation where a number of stimulus features, $\boldsymbol{x} = (x_1, x_2, \ldots, x_{n_x})$, activate a population of receptors, $\boldsymbol{s} = (s_1, s_2, \ldots, s_{n_s})$. We assume that the goal of sensory systems is to estimate the stimulus features, $\hat{\boldsymbol{x}}^{ML}$, for which the probability of the received sensory input, $\boldsymbol{s}$, is maximal. Mathematically, this means:

$$\hat{\boldsymbol{x}}^{ML} = \arg\max_{\hat{\boldsymbol{x}}} \log p\left(\boldsymbol{s}|\hat{\boldsymbol{x}}\right) \tag{1}$$

We can find $\hat{\boldsymbol{x}}^{ML}$ using a simple gradient-descent algorithm, so that each estimate is updated in time according to:

$$\frac{\partial \hat{x}_i}{\partial t} = \eta \frac{\partial \log p\left(\boldsymbol{s}|\hat{\boldsymbol{x}}\right)}{\partial \hat{x}_i}, \tag{2}$$

where η is a free parameter that determines how quickly estimates are updated. If $p\left(\boldsymbol{s}|\boldsymbol{x}\right) \propto \prod_j p\left(s_j|\boldsymbol{x}\right)$ this expression can be written:

$$\frac{\partial \hat{x}_i}{\partial t} = \eta \sum_j \frac{\partial \log p\left(s_j|\hat{\boldsymbol{x}}\right)}{\partial \hat{x}_i} \tag{3}$$

The dynamics of this algorithm depend on the generative model, $p\left(s_j|\boldsymbol{x}\right)$, describing how stimulus features in the world activate the sensory receptors. Here, we consider the case where the variance of each sensory input, $\sigma\left(\boldsymbol{x}\right)^2$, is proportional to the mean, $\mu\left(\boldsymbol{x}\right)$. The ratio between the variance and mean, $F = \frac{\sigma^2}{\mu}$, is called the Fano-factor. By chain rule, equation 3 can be written:

$$\frac{\partial \hat{x}_i}{\partial t} = \eta \sum_j \frac{\partial \mu_j\left(\hat{\boldsymbol{x}}\right)}{\partial x_i} \frac{\partial \log p\left(s_j|\mu_j\left(\hat{\boldsymbol{x}}\right), F\right)}{\partial \mu_j\left(\hat{\boldsymbol{x}}\right)}. \tag{4}$$

In the following sections we show that, for a large class of distributions with constant Fano-factor, internal estimates are updated in time according to a function of the the *ratio* between the received input, s_j, and the expected input, $\mu_j\left(\hat{\boldsymbol{x}}\right)$.

1.1 Poisson distribution

In the main text, we consider a Poisson noise, described by the probability distribution:

$$p_{\text{poiss}}\left(s|\mu\right) = \frac{1}{s!}\mu^s e^{-\mu}. \tag{5}$$

Note that for simplicity, we use simplified notation, where $\mu \equiv \mu_j\left(\hat{\boldsymbol{x}}\right)$ and $s \equiv s_j$.

For a Poisson distribution, the gradient of the log-likelihood can be written:

$$\frac{\partial \log p_{\text{poiss}}(s|\mu)}{\partial \mu} = g\left(\frac{s}{\mu}\right), \tag{6}$$

where $g(x) = x - 1$. In other words, the gradient is equal to the ratio between the received input, s, and the predicted input, μ, minus 1.

1.2 Negative binomial distribution

A generalization of the Poisson distribution, called the negative binomial distribution, allows for Fano-factors greater than 1.The negative binomial distribution can be written in the form:

$$p_{\text{neg-bin}}(s|\mu) = \frac{1}{s!}\frac{\Gamma\left(\frac{\mu}{F-1}+s\right)}{\Gamma\left(\frac{\mu}{F-1}\right)}\frac{1}{F^{\frac{\mu}{F-1}}}\left(\frac{F-1}{F}\right)^{s}. \tag{7}$$

When $F \to 1$, this distribution converges to a Poisson distribution.

If $F - 1 \ll \mu$ the gradient of the log-likelihood is closely approximated by:

$$\frac{\partial \log p_{\text{neg-bin}}(s|\mu)}{\partial \mu} \approx g\left(\frac{s}{\mu}\right) + \frac{1}{\mu}h\left(\frac{s}{\mu}\right), \tag{8}$$

where $g(x)$ and $h(x)$ are the monotonically increasing functions: $g(x) = \frac{1}{F-1}\left[\ln(1+(F-1)x) - \ln F\right]$ and $h(x) = \frac{F-1}{2F}x$. When $F \to 1$, then $h(x) \to 0$ and $g(x) \to x - 1$, so that equation 8 becomes equivalent to equation 6, obtained with the Poisson distribution

1.3 Gaussian distribution with constant Fano-factor

We now consider a gaussian distribution, with variance equal to the mean:

$$p(s|\mu) = \frac{1}{\sqrt{2\pi\mu}}e^{-\frac{(s-\mu)^2}{2F\mu}} \tag{9}$$

The gradient of the log-likelihood is:

$$\frac{\partial \log p(s|\mu)}{\partial \mu} = g\left(\frac{s}{\mu}\right) - \frac{1}{2\mu} \tag{10}$$

where $g(x) = \frac{1}{2F}\left(x^2 - 1\right)$.

When μ is large, $F \approx 1$ and x is close to 1, then equation 10 becomes equivalent to equation 6, obtained with the Poisson distribution.

1.4 Gamma distribution

Finally, we consider a gamma distribution, with variance equal to the mean:

$$p(s|\mu) = \frac{1}{\Gamma(\mu)}s^{\frac{\mu}{F}-1}e^{-\frac{s}{F}} \tag{11}$$

The gradient of the log-likelihood is:

$$\frac{\partial \log p(s|\mu)}{\partial \mu} = w_{ji}\left[g\left(\frac{s}{\mu}\right) + \frac{1}{2\mu} + C\right] \tag{12}$$

where $g(x) = \frac{1}{F}\log x$ and $C = \frac{1}{F}\log F$. As before, when μ is large, $F \approx 1$ and x is close to 1, then equation 12 becomes equivalent to equation 6, obtained with the Poisson distribution.

2 Correlated input noise

In the main text, we assumed that input noise correlations were negligible. To see how input noise correlations could influence our results, we now consider the case where the feed-forward input to the network is described by a Gaussian distribution, with signal-dependent covariance matrix $\Sigma(\mu)$:

$$\log p(\boldsymbol{s}|\boldsymbol{\mu}) \approx -\frac{1}{2}(\boldsymbol{s}-\boldsymbol{\mu})^T \Sigma(\boldsymbol{\mu})^{-1}(\boldsymbol{s}-\boldsymbol{\mu}) - \frac{1}{2}\log|\Sigma(\boldsymbol{\mu})| + \text{const.} \tag{13}$$

We consider a covariance matrix that comprises a signal-dependent part, $D(\boldsymbol{\mu})$, and a signal-independent part, Q: $\Sigma(\boldsymbol{\mu}) = D(\boldsymbol{\mu}) + \alpha Q$. The signal-dependent covariance, $D(\boldsymbol{\mu})$, is a diagonal matrix, with diagonal elements, $d_i = 1/\mu_i$.

If noise correlations are small, the log-likelihood can be approximated by a first order Taylor expansion around $\alpha = 0$:

$$\begin{aligned}\log p(\boldsymbol{s}|\boldsymbol{\mu}) \quad &\approx \quad -\frac{1}{2}\left[(\boldsymbol{s}-\boldsymbol{\mu})^T D(\boldsymbol{\mu})^{-1}(\boldsymbol{s}-\boldsymbol{\mu}) + \log|D(\boldsymbol{\mu})|\right] \\ &\quad -\alpha\frac{1}{2}\left[(\boldsymbol{s}-\boldsymbol{\mu})^T D(\boldsymbol{\mu})^{-1} Q D(\boldsymbol{\mu})^{-1}(\boldsymbol{s}-\boldsymbol{\mu}) + \boldsymbol{q}^T\boldsymbol{\mu}\right]\end{aligned} \tag{14}$$

where $\boldsymbol{q}$ is a vector consisting of the diagonal elements of Q.

The derivative is thus:

$$\begin{aligned}\frac{\partial \log p(\boldsymbol{s}|\boldsymbol{\mu})}{\partial \mu_i} \quad &= \quad \frac{1}{2}\left(z_i^2 - 1\right) - \frac{1}{2\mu_i} \\ &\quad +\alpha\left(\frac{1}{\mu_i} z_i \boldsymbol{q}_i^T(\boldsymbol{z}-1) - \frac{1}{2}q_{ii}\right) + O\left(\alpha^2\right)\end{aligned} \tag{15}$$

where $\boldsymbol{z}$ is a vector denoting the divisively normalized input ($z_i = s_i/\mu_i$), $\boldsymbol{q}_i$ is the ith column of element of Q, and q_{ii} is the ith diagonal element.

Note that the first line in equation 15 is identical to the case where there are no noise correlations (equation 10). The next line consists of a 'correction' term due to the noise correlations. In common with the first term, this term also depends on the ratio between the predicted and received feed-forward inputs, s_i/μ_i.

Thus, while input noise correlations result in quantitative changes to the steady state solution, our main result, that each feed-forward input needs to be divisively normalized by its top-down prediction, is unchanged.

3 Two-layer neural network

Here, we consider a hierarchical model in which high-level features, $\boldsymbol{y} = \left(y_1, y_2, \ldots, y_{n_y}\right)$, generate low-level features, $\boldsymbol{x} = (x_1, x_2, \ldots, x_{n_x})$, which in turn generate a sensory input, $\boldsymbol{s} =$

$\left(s_1, s_2, \ldots, s_{n_y}\right)$. For simplicity, we assume that stimulus features combine linearly, so that the mean predicted values of x_i and s_j are given by:

$$\begin{aligned} \langle s_j(\boldsymbol{x}) \rangle &= w_0 + \sum_{k=1}^{n_x} w_{jk} x_k && (16) \\ \langle x_j(\boldsymbol{y}) \rangle &= v_0 + \sum_{k=1}^{n_y} v_{jk} y_k, && (17) \end{aligned}$$

We assume that the goal of the neural network is to find maximum likelihood estimates of $\hat{\boldsymbol{x}}$ and $\hat{\boldsymbol{y}}$:

$$\begin{aligned} \{\hat{\boldsymbol{x}}_{ML}, \hat{\boldsymbol{y}}_{ML}\} &= \max_{\hat{\boldsymbol{x}}, \hat{\boldsymbol{y}}} \log p(\boldsymbol{s} | \hat{\boldsymbol{x}}, \hat{\boldsymbol{y}}) \\ &= \max_{\hat{\boldsymbol{x}}, \hat{\boldsymbol{y}}} [\log p(\boldsymbol{s} | \hat{\boldsymbol{x}}) + \log p(\hat{\boldsymbol{x}} | \hat{\boldsymbol{y}})] && (18) \end{aligned}$$

If $p(\boldsymbol{x}|\boldsymbol{y}) \propto \prod_j p(x_j|\boldsymbol{y})$, and $p(\boldsymbol{s}|\boldsymbol{x}) \propto \prod_j p(s_j|\boldsymbol{y})$, then $\hat{\boldsymbol{x}}^{ML}$ and $\hat{\boldsymbol{y}}^{ML}$ can be obtained numerically, by applying the following updates[1]:

$$\begin{aligned} \frac{\partial \hat{x}_i}{\partial t} &= \eta \left(\frac{\partial \log p(\hat{\boldsymbol{x}} | \hat{\boldsymbol{y}})}{\partial \hat{x}_i} + \sum_{j=1}^{n_s} \frac{\partial \log p(s_j | \hat{\boldsymbol{x}})}{\partial \hat{x}_i} \right) && (19) \\ \frac{\partial \hat{y}_i}{\partial t} &= \eta \sum_{j=1}^{n_x} \frac{\partial \log p(\hat{x}_j | \hat{\boldsymbol{y}})}{\partial \hat{y}_i} && (20) \end{aligned}$$

If features, $\boldsymbol{x}$, are generated via a Poisson process (i.e. $p(x_j|\boldsymbol{y}) \propto \langle x_j(\boldsymbol{y}) \rangle^{x_j} e^{\langle x_j(\boldsymbol{y}) \rangle^{x_j}}$), then equation 20 can be written:

$$\frac{\partial \hat{y}_i}{\partial t} = \eta \sum_{j=1}^{n_x} w_{ji} \left(\frac{\hat{x}_j}{\langle x_j(\hat{\boldsymbol{y}}) \rangle} - 1 \right) \quad (21)$$

which is identical to the gradient descent algorithm that we obtained with the one-layer network.

If sensory inputs, $\boldsymbol{s}$, are generated via a Poisson process (i.e. $p(s_j|\boldsymbol{x}) \propto \langle s_j(\boldsymbol{x}) \rangle^{s_j} e^{\langle s_j(\boldsymbol{x}) \rangle^{s_j}}$), then equation 19 can be written:

$$\frac{\partial \hat{x}_i}{\partial t} = \sum_j w_{ji} \left(\frac{s_j}{\langle s_j(\hat{\boldsymbol{x}}) \rangle} - 1 \right) + \left(\frac{\hat{x}_i}{\langle x_i(\boldsymbol{y}) \rangle} - 1 \right) \quad (22)$$

The first term in this expression is the same as in the one-layer network. The second-term is the fractional prediction error between the current estimate of $\hat{x}_i$, and the top-down prediction $\langle x_i(\boldsymbol{y}) \rangle$.

3.1 Neural implementation

The single-layer network described in the main text can easily be extended to a two-layer network, to implement equations 21 & 22. As with the single-layer network, each layer consists of two populations of neurons: excitatory neurons that encode the ratio between the received and predicted input ($\frac{s_j}{s_j(\hat{\boldsymbol{x}})}$ and $\frac{\hat{x}_j}{x_j(\hat{\boldsymbol{y}})}$), and inhibitory that encode the estimated stimulus features, ($\hat{x}_i$ and $\hat{y}_i$).

[1]Note that, strictly speaking x_i discrete, and thus the gradient $\frac{\partial \log p(\hat{x}_j|\hat{\boldsymbol{y}})}{\partial \hat{y}_i}$ is undefined. However, when x_i is large, it can be treated as a continuous variable with a negligible effect on inference.

The responses of neurons in the second layer are described by the following differential equations:

$$\tau_{exc}\frac{dr_{j2}^{exc}}{dt} = I_j - \left(v_0 + \sum_k v_{jk} r_{k2}^{inh}\right) r_{j2}^{exc} \tag{23}$$

$$\tau_{inh}\frac{dr_{i2}^{inh}}{dt} = \sum_j v_{ji}\left(r_{j2}^{exc} - 1\right), \tag{24}$$

where I_j represents the input to the jth excitatory neuron in the 2nd-layer, which evolves in time according to equation 26 (so that $I_j = r_{j1}^{inh}$). The response of each neuron is labelled with two indices: for example, r_{jk}^{exc} corresponds to the response of the jth excitatory neuron in the kth layer of the network.

The responses of neurons in the first layer of the network are described by the following differential equations:

$$\tau_{exc}\frac{dr_{j1}^{exc}}{dt} = s_j - \left(w_0 + \sum_k w_{jk} r_{k1}^{inh}\right) r_{j1}^{exc} \tag{25}$$

$$\tau_{inh}\frac{dr_{i1}^{inh}}{dt} = \sum_j w_{ji}\left(r_{j1}^{exc} - 1\right) - \left(r_{i2}^{exc} - 1\right). \tag{26}$$

These equations are very similar to the one layer network, described in the main text (main text, equations 5 & 6), with the addition of a second term in equation 26, that denotes feed-back from downstream neurons.

4 Analytical expression for neural firing rates

In this section, we show that the canonical normalization model of Heeger et al. emerges as a special case of our model.

In the steady state, the response of the jth excitatory neuron is given by:

$$r_j^{exc} = \frac{s_j}{w_0 + \sum w_{jk} r_k^{inh}}. \tag{27}$$

Substituting this into the equation 6 in the main text, and setting $\frac{\partial r_i^{int}}{\partial t} = 0$, we obtain:

$$0 = \sum_j w_{ji}\left(\frac{s_j}{w_0 + \sum_k w_{jk} r_k^{inh}} - 1\right). \tag{28}$$

In the general case where there are many excitatory and inhibitory neurons, equation 28 cannot be solved exactly. However, in the special case where, for all $k \neq i$, either $\sum_j w_{ji} w_{jk} s_j = 0$ or $r_k^{int} = 0$, equation 28 simplifies to:

$$0 = \sum_j w_{ji}\left(\frac{s_j}{w_0 + w_{ji} r_l^{inh}} - 1\right). \tag{29}$$

If $w_0 \ll w_{ji} r_l^{inh}$, the above equation can be solved, to give,

$$r_i^{inh} \approx \frac{\sum_j (s_j - w_0)}{\sum_j w_{ji}}. \tag{30}$$

Substituting this expression for r_i^{inh} back into equation 27, we obtain a closed-form expression for the excitatory neuron response:

$$r_j^{exc} = \frac{s_j}{\left|\tilde{w}_{ji}\sum_j s_j\right|}, \quad (31)$$

where $\tilde{w}_{ji} = \frac{1}{\sum_j w_{jl}} w_{jl}$, $|x| = \max(x, w_0)$, and $i = \arg\max_k \sum_j s_j \log\left(\tilde{w}_{jk}\right)$.

Equation 31 is of a very similar form to the canonical normalization model proposed by Heeger et al., in which neural responses are described by the equation:

$$r_j^{exc} = \gamma \frac{I_j^n}{\sigma_j^n + \sum_j I_j^n}, \quad (32)$$

where I_j is the j^{th}input to the network, and γ, n & σ are free parameters that determine the shape of the contrast response curve.

One can show that in order for neural responses to be well approximated by equation 31, the stimulus should not drive the inputs to more than one competing interneuron too strongly. Mathematically, we require that for all $k \neq i$,

$$\sum_j \frac{\tilde{w}_{jk}}{\tilde{w}_{ji}} \tilde{s}_j \quad < \quad 1 \quad \text{or} \quad \sum_j \tilde{w}_{jk}\tilde{w}_{ji}s_j = 0, \quad (33)$$

where $\tilde{s}_j = \frac{1}{\sum_j s_j} s_j$ and $\tilde{w}_{ji} = \frac{1}{\sum_j w_{ji}} w_{ji}$.

Supplementary figure 1 shows the response of a single excitatory neuron versus the strength of input to the network, with the excitatory response computed numerically (solid lines) or using equation 31 (see figure legend for simulation details). As can be seen in this figure, equation 31 provides a good description of the neuron's response.

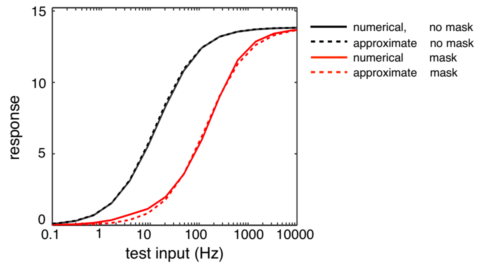

Figure 1: Comparison between simulation and analytic results. The network consists of 30 inhibitory and 30 excitatory neurons. Connection strengths between inhibitory and excitatory neurons are chosen from a uniform distribution between 0 and 40. In the 'no mask' condition one of the sensory inputs varies between 10^{-1}Hz and 10^4Hz, while all the other inputs are set to the background input of $w_0 = 1$Hz. In the 'mask condition' all sensory inputs are activated at 10Hz. Solid lines plot the steady-state response of the maximally driven excitatory neuron, in the 'no mask' and 'mask' conditions. Dashed curves show the response of the neuron, approximated using supplementary equation 31.

5 Comparison to a global divisive inhibition model

In figures 2-5 of the main text, we compared the divisive-input model (predicted by optimal estimation with signal-dependent noise) to an LN model, where responses are obtained by

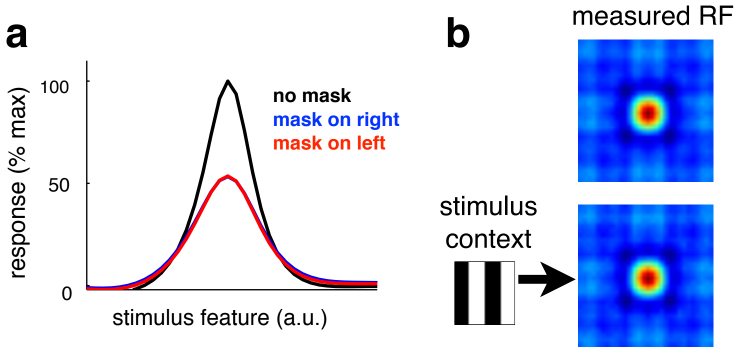

Figure 2: Predictions of global divisive inhibition model. The figure is the same as figure 2c-d and in the main text, but with a global divisive inhibition model (with responses described by supplementary equation 34).

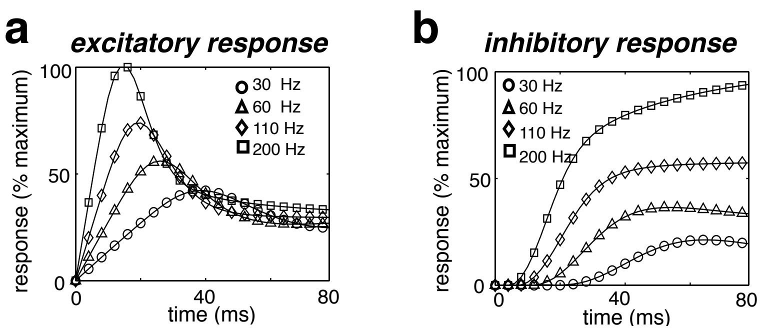

Figure 3: Effect of varying excitatory/inhibitory timescales. The figure is the same to figure 7a-b in the main text, with the exception that the timescale of inhibition has been increased by a factor of 40, relative to excitation.

linearly integration of inputs followed by non-linear rescaling of responses. This LN model is a simple generalisaion of a subtractive inhibition model (predicted by optimal estimation with gaussian noise), which predicts linear responses. It is straight-forward to show that such an LN model is incapable of producing contextual tuning curve shifts, regardless of the form of the non-linearity.

In addition, we compared our model to a global-divisive model, with responses were described by the following equation:

$$r_i = \frac{\sum_j U_{ji} s_j}{\sum V_{ji} s_j + C} \tag{34}$$

where U_{ji} and V_{ji} are positive input and divisive filters, and $|\cdot|$ is a rectifying non-linearity. As with the LN model shown in the main text, linear filters, U_{ji} and V_{ji}, were learned in each case so as to minimise the mean squared difference between the responses obtained with the input-targeted inhibition model in the main text and the above global divisive inhibition model.

Supplementary figures 2a & b show the responses obtained with global divisive inhibition, with paramaters trained to reproduce the tuning curves shown in figure 2c-d in the main text. As can be seen, global divisive inhibition was unable to produce contextual shifts in tuning curves.

6 Temporal dynamics of neural responses

In figure 7 we plotted the temporal response profiles of excitatory and inhibitory neurons in our model, in response to a constant input. To investigate how our results depended on the relative timescale of excitation and inhibition, we ran an additional simulations with the relative timescale of excitation increased by a factor of 40, relative to the timescale of inhibibion (i.e. determined ratio between a and b in equations 5-6 in the main text; Supp. fig. 3)

Increasing the relative speed of excitation resulted in more pronounced input-dependent variations in the time that excitory responses took to reach their peak (Supp. fig. 3a). Also, inhibitory responses were quicker to arrive at steady-state, and in some cases showed a transient overshoot (Supp fig 3b). Overall, however we observed qualitatively similar results to figure 7a-b, in the main text.
