# Supplementary figures and images for: Sensory noise predicts divisive reshaping of receptive fields
Source: PLoS Comput Biol. 2017 Jun 16;13(6):e1005582. doi: 10.1371/journal.pcbi.1005582 (PMC5509365; doi:10.1371/journal.pcbi.1005582)

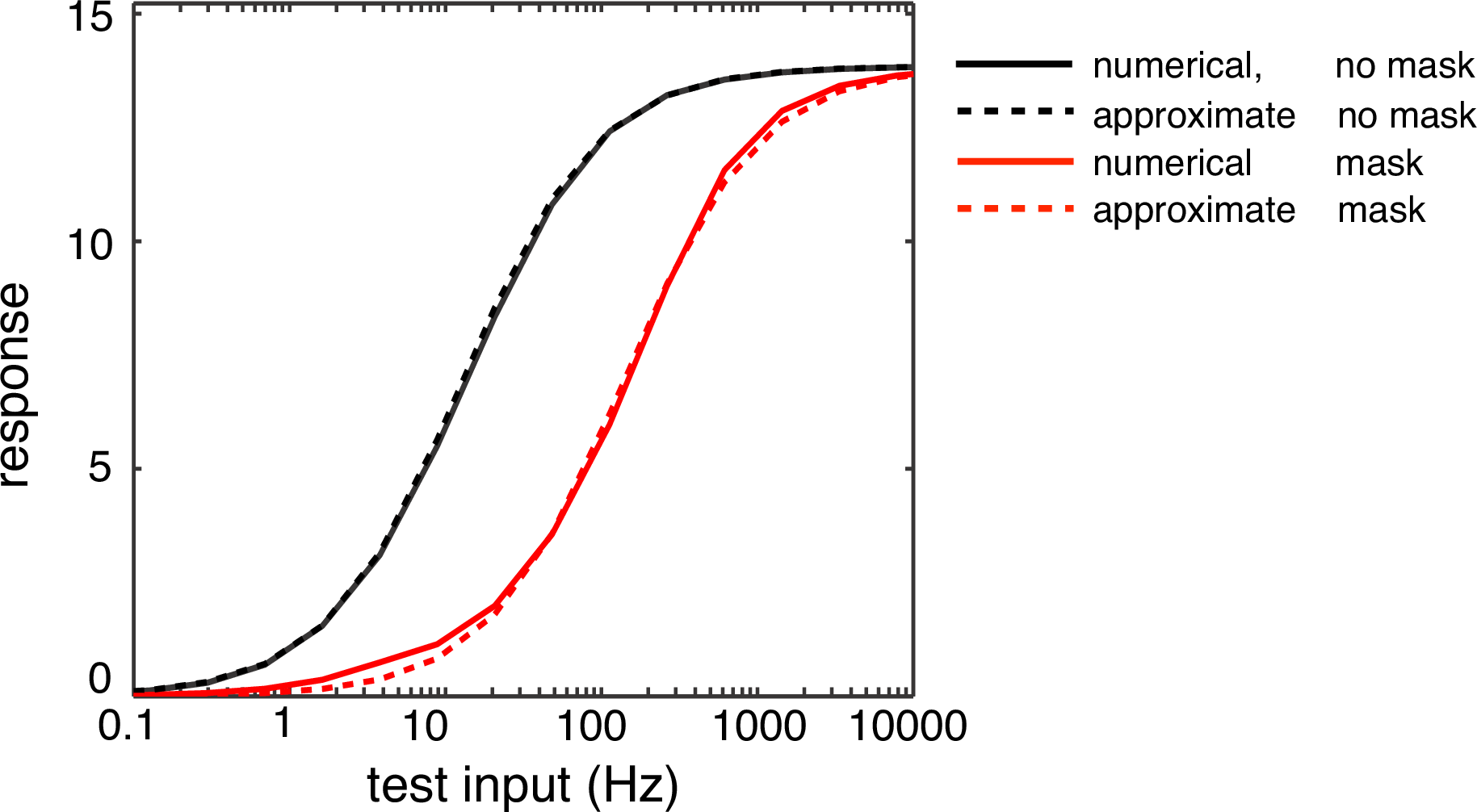

Supplement: S1 Fig — The network consists of 30 inhibitory and 30 excitatory neurons. Connection strengths between inhibitory and excitatory neurons are chosen from a uniform distribution between 0 and 40. In the ‘no mask’ condition one of the sensory inputs varies between 10−1Hz and 104Hz, while all the other inputs are set to the background input of w0 = 1Hz. In the ‘mask condition’ all sensory inputs are activated at 10Hz. Solid lines plot the steady-state response of the maximally driven excitatory neuron, in the ‘no mask’ and ‘mask’ conditions. Dashed curves show the response of the neuron, approximated using eq 31 in S1 Text. (TIF) [file pcbi.1005582.s002.tif]

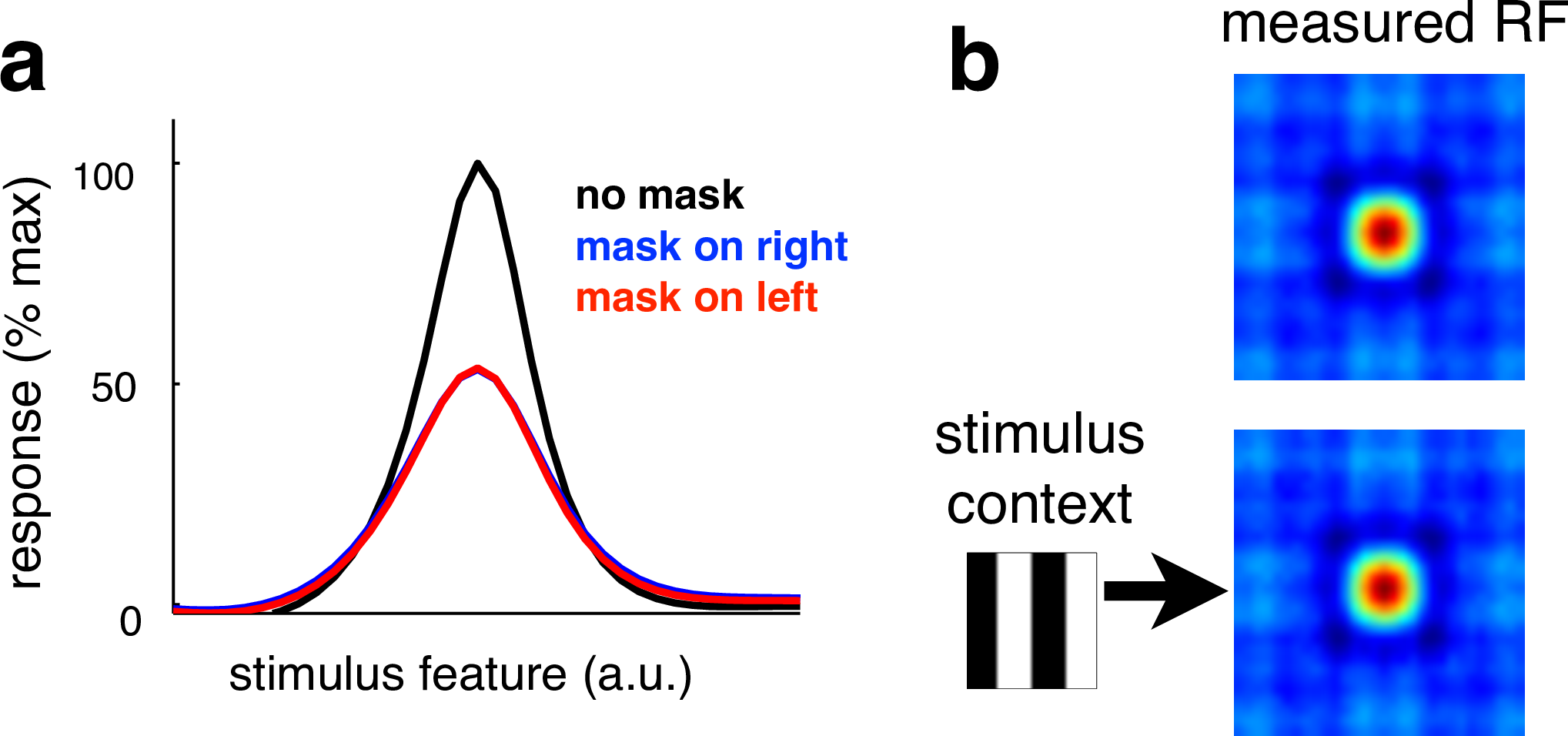

Supplement: S2 Fig — The figure is the same as Fig 2c and 2d and in the main text, but with a global divisive inhibition (with responses described by eq 34 in S1 Text). (TIF) [file pcbi.1005582.s003.tif]

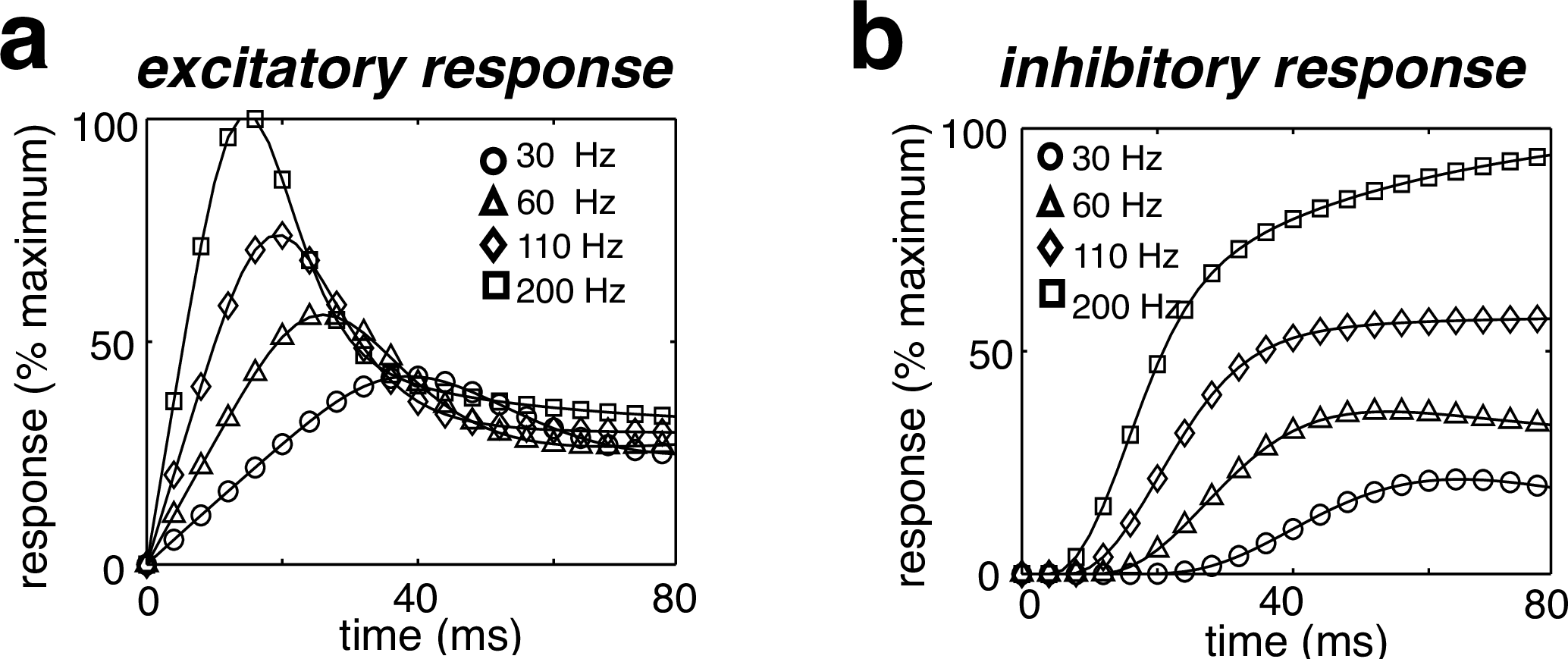

Supplement: S3 Fig — The figure is the same to Fig 7a and 7b in the main text, with the exception that the timescale of inhibition has been increased by a factor of 40, relative to excitation. (TIF) [file pcbi.1005582.s004.tif]
